# Cropping practices manipulate abundance patterns of root and soil microbiome members paving the way to smart farming

**DOI:** 10.1186/s40168-017-0389-9

**Published:** 2018-01-16

**Authors:** Kyle Hartman, Marcel G. A. van der Heijden, Raphaël A. Wittwer, Samiran Banerjee, Jean-Claude Walser, Klaus Schlaeppi

**Affiliations:** 1 Plant-Soil Interactions, Department of Agroecology and Environment, Agroscope, Zurich, Switzerland; 2 Institute for Evolutionary Biology and Environmental Studies, University of Zurich, Zurich, Switzerland; 3 Genetic Diversity Centre, ETH Zurich, Zurich, Switzerland; 4 Plant-Microbe Interactions, Institute of Environmental Biology, Faculty of Science, Utrecht University, Utrecht, The Netherlands

**Keywords:** Soil and root microbiomes, Microbial co-occurrence, Network analysis, Cropping practices, Microbiota management, Smart farming

## Abstract

**Background:**

Harnessing beneficial microbes presents a promising strategy to optimize plant growth and agricultural sustainability. Little is known to which extent and how specifically soil and plant microbiomes can be manipulated through different cropping practices. Here, we investigated soil and wheat root microbial communities in a cropping system experiment consisting of conventional and organic managements, both with different tillage intensities.

**Results:**

While microbial richness was marginally affected, we found pronounced cropping effects on community composition, which were specific for the respective microbiomes. Soil bacterial communities were primarily structured by tillage, whereas soil fungal communities responded mainly to management type with additional effects by tillage. In roots, management type was also the driving factor for bacteria but not for fungi, which were generally determined by changes in tillage intensity. To quantify an “effect size” for microbiota manipulation, we found that about 10% of variation in microbial communities was explained by the tested cropping practices. Cropping sensitive microbes were taxonomically diverse, and they responded in guilds of taxa to the specific practices. These microbes also included frequent community members or members co-occurring with many other microbes in the community, suggesting that cropping practices may allow manipulation of influential community members.

**Conclusions:**

Understanding the abundance patterns of cropping sensitive microbes presents the basis towards developing microbiota management strategies for *smart* farming. For future targeted microbiota management—e.g., to foster certain microbes with specific agricultural practices—a next step will be to identify the functional traits of the cropping sensitive microbes.

**Electronic supplementary material:**

The online version of this article (10.1186/s40168-017-0389-9) contains supplementary material, which is available to authorized users.

## Background

Agricultural intensification has resulted in an increased production of staple crops such as wheat, rice, and maize and lead to greater food security for a continuously growing world population [1, 2]. Despite these benefits, there is increasing awareness about the adverse environmental impacts arising from the intensive practices of modern agriculture. These include increased greenhouse gas emissions and nutrient leaching as a result of intensive fertilizer application [3], increased soil erosion [4], and detrimental effects on biodiversity [5, 6]. To alleviate such deleterious effects, an ecological intensification has been proposed that focuses on meeting standards of environmental quality while promoting and maintaining organisms that provide beneficial ecosystem services [7, 8]. A number of practices improve the sustainability of agriculture, including organic farming [9] and reduced or no-tillage [10]. These practices aim to enhance soil fertility while maintaining crop yields through supporting a diverse and active soil biota [11]. Soil biota includes microbes such as bacteria and fungi that collectively function as a microbiome. Bacteria and fungi regulate many ecosystem processes and play key roles in nutrient cycling through decomposition of organic matter, and transformation and fixation of important soil nutrients like nitrogen and phosphorus [12].

Aside from the environmental benefits of organic agriculture [13] and less intensive tillage regimes [10], there is still debate about the effects of these cropping practices on belowground microbial communities. In general, arable management affects community composition and diversity; although such effects may depend on the microbial kingdom being studied and the different farming systems being compared [14, 15]. However, there are few agricultural experiments comparing conventional and organic farming practices [16] and fewer that compare different management types and tillage intensities [17]. Therefore, an agricultural experiment combining these two aspects at a single site allows to separate the effects of management type and tillage on microbial communities and minimize variation caused by soil spatial heterogeneity. The Farming System and Tillage experiment (FAST) was established in 2009 near Zürich to address this for the main arable cropping systems in Switzerland (Additional file 1: Fig. S1). These cropping systems are, namely, conventional (C) and organic (O) management types, with different tillage intensities (no-tillage (NT), reduced-tillage (RT), and intensive tillage (IT)). The FAST experiment compares the 4 main cropping systems C-IT, C-NT, O-IT, O-RT. Wittwer et al. [18] described the design of the FAST experiment and provided agronomic insights into the effects of different farming practices on winter wheat and maize yields. However, the effects of management types and different tillage intensities on belowground microbial communities at the site have not been explored.

Soil microbial communities and their associated functions largely determine the productivity of agroecosystems [19]. The composition of the soil microbiome presents the major driver in shaping the bacterial and fungal communities associated with plant roots [20, 21]. Root microbiome is an important determinant for plant growth and health by assisting in nutrient uptake, supporting abiotic stress tolerance and protecting the host from pathogens [20, 22]. A number of recent microbial community surveys have described the root microbiomes of *Arabidopsis thaliana* [23, 24], clover [25], maize [26], rice [27], sugarcane [28], and grapevine [29] and reported significant effects of soil type on root microbiome composition. If soil and root microbial communities are closely linked, root microbial communities may also be affected by agronomic practices [30]. To date, the effects of agricultural practices on root microbial communities remain still poorly understood, owing to contrasting reports and the use of low-resolution fingerprinting methods [31]. Using high-throughput sequencing, we aimed to unravel how root microbial communities respond to conventional and organic agriculture and various tillage regimes.

Members of the soil and root microbiome interact directly and indirectly with each other, and a tool for better understanding of these potential interactions is co-occurrence network analysis [32, 33]. Long used in the social sciences to analyze relationships between humans [34], network analyses have recently been applied in soil microbial ecology to explore patterns of community assembly [35], visualize response patterns of different taxonomic groups to agronomic practices [36], and to identify individual microbiome members that significantly influence community composition [37]. It was recently shown that soils under conventional and organic management harbor distinct microbial networks in each farming system [38]. To date, the effects of different cropping practices on co-occurrence patterns in the root microbiome remain unexplored.

From the perspective of microbiome management, it is important to understand which microbes are sensitive to cropping practices and whether they possess specific network properties. Microbes that frequently co-occur with many others are referred to as keystone taxa because they may play an ecologically important role by determining community dynamics and microbiome functioning [37–39]. It is unclear whether keystone taxa in soil and root microbiomes are responsive to cropping practices. More importantly, are cropping sensitive microbes solitary community members, or do they belong to guilds of simultaneously responding taxa? Are they frequent or not? Such information is relevant for implementing agricultural management strategies to promote specific microbes that contribute to soil fertility and plant health.

With these ideas in mind, we investigated the impact of cropping practices at the FAST experimental site on soil and root bacterial and fungal communities in winter wheat using amplicon sequencing and network analysis. We specifically asked: (1) Do soil and root microbial communities differ in their responses to management type and tillage intensities? (2) Which microbes are the indicator taxa for particular cropping practices (conventional vs. organic; reduced vs. intensive tillage)? (3) How do cropping practices impact co-occurrence patterns of soil- and root-associated microbes? (4) What are the network characteristics (abundance, degree of co-occurrence, and keystoneness) of cropping sensitive microbes?

## Results

### Soil and root microbiota

We conducted separate bacterial and fungal community profiling of 16 soil and 16 winter wheat root samples from of the FAST experiment (Additional file 1: Fig. S1) to investigate the effects of management type and tillage intensity on microbial communities. The bacterial community profiling yielded a total of 639,440 high-quality sequences (range 11,192–37,255; median 18,122; Additional file 2). Fungal profiling yielded 962,619 sequences, ranging between 9138 and 48,750 sequences per sample (median 30,284). We identified 2972 bacterial, 3 archaeal, and 1975 fungal operational taxonomic units (OTUs) across all samples (Additional file 1: Fig. S2).

**Fig. 1:**
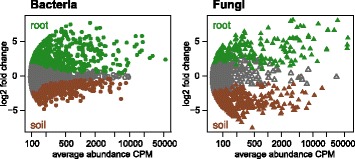
Soil and roots harbor specific sets of microbes. MA plots displaying the abundance patterns of bacteria and fungi in soil and root microbiomes. *X*-axis reports average OTU abundance (as counts per million, CPM), and *Y*-axis log2-fold change (root relative to soil). Root and soil-specific OTUs were colored in green and brown, respectively, and non-differentially abundant OTUs are in gray (likelihood ratio test, *p* < 0.05, FDR corrected)

Plant roots and soil present different microbial habitats with specific sets of microbes (Fig. 1). Taxonomies are described in the supplement (Additional file 1: Supplementary Results and Fig. S3). We visualized and quantified the differences between microbial communities (β-diversity) using unconstrained principal coordinate analysis (PCoA) and permutational multivariate analysis of variance (PERMANOVA) on Bray-Curtis dissimilarities. Microbial communities of root and soil clearly separated along axis 1 (Additional file 1: Fig. S4). The discrete outlier in the bacterial communities was consistent with relatively low soil pH in one subplot. We only noted a subtle clustering by cropping practices along axis 2 where the root fungi tended to group by the intensity of tillage. PERMANOVA confirmed the marked differences between the two microbial habitats (bacteria *R*^2^ = 0.602, *P* < 0.001; fungi *R*^2^ = 0.376, *P* < 0.001) and smaller but significant impact of cropping practices (bacteria *R*^2^ = 0.086, *P* < 0.05; fungi *R*^2^ = 0.102, *P* < 0.05; Additional file 1: Table S2).

For α-diversity analyses, we rarified the communities to 11,000 (bacteria) and 9000 (fungi) sequences per sample, which captured most of the observed OTU richness (Additional file 1: Fig. S5). Soils supported higher species richness than roots with bacterial communities being greater in richness than fungi (Additional file 1: Fig. S5 and Table S3). In both soil and root communities, bacteria and fungi richness was highest in O-IT samples with significant effect for bacterial communities in root samples. To conclude, plant root and soil microbiota differ markedly in richness, composition, and taxonomy.

### Cropping system effects on soil and root microbial communities

**Fig. 2:**
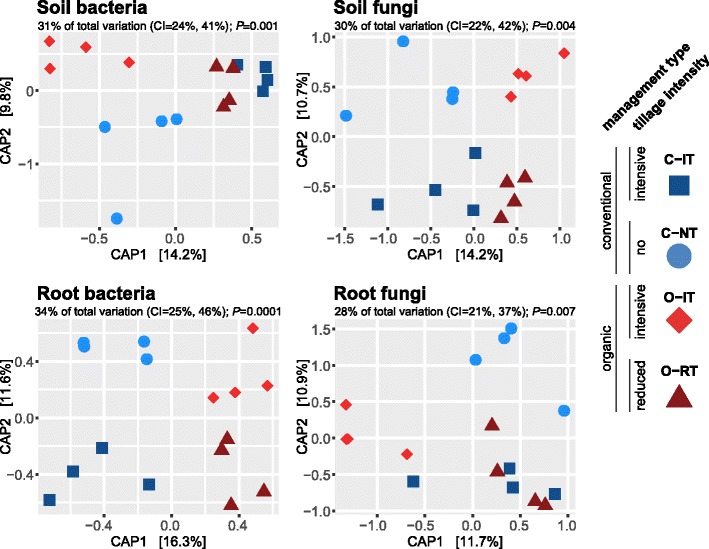
Effects of cropping practices on soil and root bacteria and fungi. Separate CAP ordinations using Bray-Curtis distance were performed for bacteria and fungi, both in roots and soil. CAP analyses were constrained by the factor “cropping systems”, and the explained fraction of the total variance is indicated above the plots (with 95% confidence interval, significance assessed with 10^4^ permutations). Percentage of variation given on each axis refers to the explained fraction of total variation

For in-depth analysis of cropping system effects on root and soil microbial communities, we employed canonical analysis of principal coordinates (CAP). Partial CAP—constrained by cropping system—highlighted a tillage effect on soil bacteria and both management and tillage effects on soil fungal communities (Fig. 2). PERMANOVA confirmed the significant effect of cropping systems on both soil microbial communities (Additional file 1: Table S4). Pairwise tests revealed significant differences between the two conventional and O-RT treatments but not O-IT treatments for soil bacteria. For the soil fungi, significant differences were found between the low-intensity tillage treatments and O-IT but not C-IT treatments.

Different patterns were observed for the root microbiota. Root bacteria formed four distinct clusters in the ordination with axis 1 again separated the samples by management type and axis 2 separated the samples by tillage intensity. Pairwise PERMANOVA comparisons detected significant differences between the two conventional treatments and O-RT but not O-IT samples. For root fungal communities, CAP separated the O-RT samples along axis 1 and the C-NT samples from the other treatments on axis 2. PERMANOVA also confirmed a general effect of cropping system, but no pairwise differences on community dissimilarity were found.

Since β-diversity can be driven by true biological differences, differences in group dispersion (variance), or both [40], we tested for differences in dispersion for both soil and root microbiota using BETADISP. The lack of significance in these dispersion tests suggested that differences between cropping systems were driven primarily by true biological differences and not an artifact of differences of within-group dispersion (Additional file 1: Table S4). In summary, while tillage-driven differences were seen in the soil bacterial community, the management type appeared to be the main driving factor in root bacteria. Conversely, root fungal communities did not strongly respond to management type induced changes in soil and instead were determined by changes in the tillage intensity.

### Identifying cropping sensitive OTUs

**Fig. 3:**
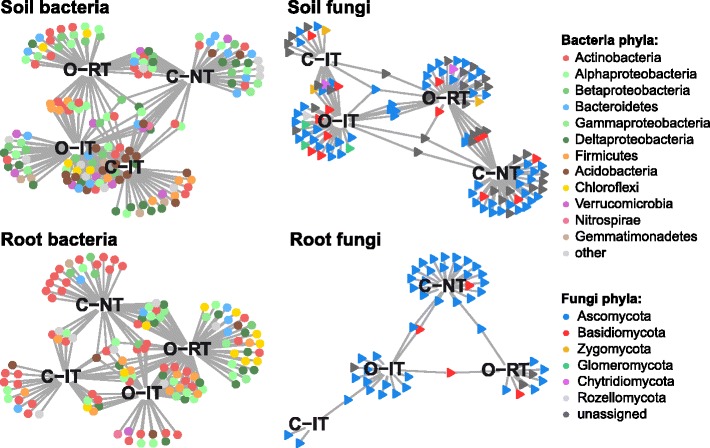
Bipartite networks display cropping system specific OTUs in the soil and root bacterial and fungal communities as determined using indicator species analysis. Circles represent individual bacteria and triangles fungi OTUs that are positively and significantly associated (*p* < 0.05) with one or more of the cropping systems (association(s) given by connecting lines). OTUs are colored according to their Phylum assignment

We employed indicator species analysis to identify individual bacteria (bOTUs) and fungi (fOTUs) in soil and root communities whose abundances varied between the different cropping systems, and we summarized the analysis with a bipartite network (Fig. 3; Additional file 3). Patterns were reminiscent of the effects seen in the previous diversity analyses. For instance, the high number of soil bacteria OTUs that were shared between the intensive tillage reflects the close clustering of these samples in the ordination. Similarly, consistent with the finding that both management type and tillage intensity explain variation among soil fungi, we found high numbers of indicator OTUs specific to one-cropping system.

As indicator OTUs were solely identified based on correlation, we validated them using likelihood ratio tests implemented in edgeR ([41]; Additional file 3). Finally, we defined the OTUs that were supported by both methods as cropping sensitive OTUs (hereafter: *cs*OTUs). In soil, we found a total of 53 and 70 bacteria and fungi *cs*OTUs, respectively (Additional file 1: Fig. S6). As approximation for an “effect size” of cropping practices on microbial communities, we calculated these bacteria and fungi *cs*OTUs to account for 8.3 and 9.9% of the total soil community sequences. Similarly, we identified 62 and 36 *cs*OTUs for root bacteria and fungi, corresponding to an effects size of 14.2 and 5.0%, respectively. Consistent with the previous conclusion that cropping practices affected soil and root communities differently, we saw little overlap between bacteria and fungi *cs*OTUs comparing root and soil samples. While the identified *cs*OTUs responded to specific cropping systems, they did not exhibit a particular taxonomic pattern with cropping system (Additional file 1: Figs. S7-S12 and Supplementary Results). Taken together, each cropping system supports a specialized subset of soil and root bacteria and fungi, while the majority of the communities are shared between management types and tillage regimes.

### Cropping effects on microbial co-occurrence patterns

Lastly, we explored the extent to which management types and tillage regimes impacted co-occurrence patterns in microbial communities. We first constructed separate co-occurrence networks for soil and root bacterial and fungal communities and determined their properties (see the “Methods” section). Consistent with the α-diversity analyses (Additional file 1: Fig. S5), the soil bacteria network comprised the highest number of significantly co-occurring OTUs, followed by intermediate and similar numbers in the soil fungi and root bacteria networks (Additional file 1: Fig. S13). Consequently, network connectivity (measured by average number of connections per OTU) was higher in the soil bacteria and soil fungi networks than the root bacteria network. The root fungi network comprised the fewest OTUs and was the least complex. We also mapped the *cs*OTUs (as defined in Additional file 1: Fig. S6) into the microbial networks, and we found them agglomerating according to management type and/or tillage intensity (Additional file 1: Fig. S13).

**Fig. 4:**
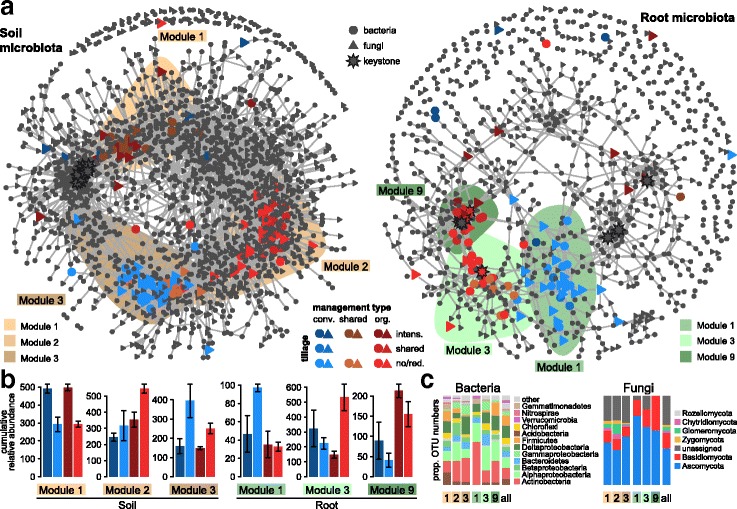
Co-occurrence patterns of cropping sensitive OTUs. **a** Co-occurrence networks visualizing significant correlations (*ρ* > 0.7, *p <* 0.001; indicated with gray lines) between bacteria and fungi OTUs in soil and root communities. Circles indicate bacteria, triangles fungi, and keystone OTUs are represented with asterisks (Table 1). OTUs are colored by their association to the different cropping systems (as defined in Additional file 1: Figure S6; gray OTUs are insensitive to cropping practices). Shaded areas represent the network modules containing *cs*OTUs as defined in Additional file 1: Figure S14. **b** Cumulative relative abundance (as counts per million, CPM; *y*-axis in ×1000) of all bacteria and fungi of the cropping sensitive modules in soil and root networks. The cumulative relative abundance in samples of C-IT (dark blue), C-NT (light blue), O-IT (dark red), O-RT (light red) cropping systems indicates the overall response of cropping sensitive modules to the different farming practices. **c** Qualitative taxonomic composition of cropping sensitive modules is reported as proportional OTUs numbers per class (bacteria) and phylum (fungi) and compared to the overall taxonomic distribution in the entire dataset (column “all”)

Next, we explored the distribution patterns of *cs*OTUs in meta co-occurrence patterns of bacteria and fungi in soil and root communities (Fig. 4a, Table 1). We found that the abundance patterns of inter-kingdom microbial associations also responded to cropping practices. We noted in the soil and root meta-networks that three modules contained relatively high proportions of *cs*OTUs (Additional file 1: Fig. S14; Additional file 3). The type of sensitivity of these module members to the specific cropping systems (Fig. 4b) and their distribution in the network partially reflected the drivers of community dissimilarity seen in the CAP ordinations (Fig. 2). For example, the effect of tillage intensity in the soil communities was apparent with a discrete module (M1) in the soil network, containing *cs*OTUs specific to intensive tillage practices. M1 was separated from two other modules (M2 and M3) that primarily contained *cs*OTUs specific to the O-RT and C-NT cropping systems (Fig. 4a, b). Similarly, management type presented the main driver in root communities (Fig. 2), and the numerous *cs*OTUs assigned to organic management were predominantly located in modules M3 and M9 and separated from module M1 containing primarily conventional management specific OTUs (Fig. 4b). Furthermore, the separation of the two modules containing *cs*OTUs specific to organic production systems appeared to reflect differences in tillage practices (Fig. 4a, b). All the management and tillage responsive modules in soil and roots comprised a taxonomically broad set of bacteria and fungi (Fig. 4c), revealing that the different cropping practices do not target specific microbial lineages.

**Table 1:** Properties of soil and root meta co-occurrence networks

Community	^a^OTUs		^b^Connections			^c^Connectivity	^d^Keystone		^e^*cs*OTUs	
	Bacteria	Fungi	Bac-Bac	Fun-Fun	Bac-Fun	Network wide	Bacteria	Fungi	Bacteria	Fungi
Soil	1197	747	1904	1111	2270	5.4	10	9	51 (0)	69 (0)
Root	688	239	855	159	434	3.1	9	0	57 (5)	33 (0)

^a^Number of network nodes

^b^Number of network edges

^c^Mean number of connections per node

^d^Number of keystone OTUs

^e^Number of cropping sensitive OTUs present in the network (number of keystone OTUs therein)

**Fig. 5:**
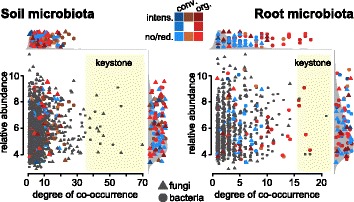
Degree of co-occurrence and abundance of *cs*OTUs. Relative abundance (as counts per million, CPM) of all OTUs from the soil and root microbiome co-occurrence networks (Fig. 4) was plotted as a function of their degree of co-occurrence. Circles and triangles refer to bacteria and fungi, respectively. OTUs were colored by their association to the different cropping systems and keystone OTUs (top 1% degree of co-occurrence) have yellow background. Side panels recapitulate the distributions of co-occurrence degrees and abundance for the *cs*OTUs (shapes colored by association to cropping systems) compared to the density of all

The *cs*OTUs were identified among low count as well as among highly abundant soil and root taxa (Fig. 5). In soil, they had low to medium degrees of co-occurrence, while in roots they were also found among OTUs that co-occurred with many other taxa. In roots, we observed that “organic” *cs*OTUs exhibited higher degrees of co-occurrence than “conventional” *cs*OTUs. With the exception of five root bacteria OTUs, the majority of keystone OTUs was not sensitive to cropping practices (Table 1**,** Additional file 1: Table S5). The keystone *cs*OTUs were from the Firmicutes (*bOTU23*, *bOTU119* family *Peptostreptococcaceae*, *bOTU36* family *Erysipelotrichaceae*), the Chloroflexi (*bOTU949*, family *Chloroflexaceae*), and the Actinobacteria (*bOTU530* family *Microbacteriaceae*) and had higher abundances in roots from organically managed plots (Additional file 1: Fig. S11).

Taken together, we show that the differing cropping practices markedly alter co-occurrence patterns of numerous bacteria and fungi, and these impacts affected microbiome members largely independent of their abundance and connectivity.

## Discussion

While considering the effects of agricultural practices on soil microbial communities, previous studies have often been limited to the examination of single factors like management type [42–44], tillage [45–47], soil amendments [48–50], or focused on either the bacterial or fungal kingdom. Hartmann et al. [36] investigated effects of management type on soil bacteria and fungi in a multi-factor long-term agricultural experiment. Our motivation was to expand on these efforts by examining cropping system effects on *root* microbes and by also including the effect of tillage into the experimental design. To our knowledge, this is the first study to investigate how conventional and organic managements, both with different tillage regimes, influence bacterial and fungal communities in both soil and root compartments. The strength of the FAST experiment is that management and tillage effects can be studied factorially and independent of possibly confounding variables such as soil types. However, a broader generalization of the presented findings requires further studies with greater sample numbers, from multiple sites, across different climatic conditions and accounting for temporal and special variation in root and soil microbiomes.

### Differential responses of soil and root microbiomes

The specific sets of microbes in roots and soil (Fig. 1) explain the clear separation by compartment in the ordination analyses (Additional file 1: Fig. S4). This distinctiveness of the plant root microbiome was also found in previous studies of other plant species [23–26, 28, 29, 51]. Plants recruit a root microbiome in their early life stages from a larger pool of soil microbes [27, 52]. The initial composition of this soil microbial pool is the most influential factor determining the composition of root microbial communities [23, 24, 53, 54]. Therefore, we also investigated if root bacterial and fungal communities reflected cropping system-driven differences in soil microbial communities (see Additional file 1: Supplementary Discussion).

We observed compartment-specific responses of bacteria and fungi to the tested cropping practices (Fig. 2, Additional file 1: Table S4). Dissimilarities in soil bacteria were generally driven by differences in tillage regimes, whereas management type, together with tillage, was influential for the soil fungi. Notably, the most influential cropping factors driving differences in soil communities were not necessarily the most influential in root communities. In the root microbiome, we found that the management type was the most influential factor for the root bacteria, while tillage intensity explained most of the variation in the root fungi. Taken together, our results demonstrate that agricultural management affects soil and root microbial communities differently.

We hypothesize that a combination of timing and nutrient characteristics of root and soil compartments could explain the differential responses of soil and root microbiomes to the cropping practices. By timing, we refer to the different time points between the effective cropping practices (mostly before seeding) and the harvest or sampling of the crop. We assume that the soil microbiome would exhibit the most pronounced differences in response to tillage or manure fertilization shortly after application and that such effects would gradually decline over the length of the growing season until a soil-type specific equilibrium is reached again after disturbance. For the root microbiome, however, crops are sown soon after tillage or manure application and the roots recruit microbes from the most divergent conditions so that the pronounced differences between cropping practices may be “fixed” for a longer time. Hence, primary colonizers that coin the root microbiome assembly at early stages would explain the preservation of precedent management differences. In addition, the nutritional characteristics of the root compartment may contribute to preserving specific management differences. Compared to an oligotrophic soil environment, we consider plant roots a copiotrophic compartment due to the continuous secretion of root exudates. Nutrient-rich organic fertilizers mainly contain copiotrophic gut bacteria from cattle that may also find favorable conditions in the root compartment. We see support for this idea as there is a marked impact of organic management on the root bacteria and not on soil (Figs. 2 and 3) and because the only bacteria with high degrees of co-occurrence were exclusively found in the root microbiome networks and were specific to organic farming (Fig. 5). Future experiments are needed to test these hypotheses. Such experiments would include, e.g., the quantification of cropping-induced microbiome differences of soil and root samples in time series throughout the growing season or manure application tests that uncouple nutritional from microbial components (e.g., applications of nutrient-free microbial extracts from slurry or extracts with inactivated slurry microbiota).

We confirmed the soil to be more diverse than the root microbial communities [20, 55]; however, we only found marginal impacts of the cropping systems on bacteria and fungi α-diversity (Additional file 1: Fig. S5 and Table S3, see Supplementary Discussion). Hence, the different cropping practices affected species richness to smaller degree than community composition. This is consistent with previous observations that species richness was less variable in their responses to environmental factors (i.e., different cropping systems) than species composition [56, 57]. Changes in microbial community composition may not necessarily lead to altered diversity or richness because changes of some taxonomic groups may be compensated by changes in others [57] and because univariate measures of diversity and richness mask relationships between individual and groups of taxa [58].

### Cropping sensitive microbes

We identified cropping sensitive *cs*OTUs in both soil and root microbial communities (Additional file 1: Fig. S6), and they function as indicator taxa to explain the β-diversity patterns by cropping practices (Fig. 2). For example, the higher relative abundance of bacteria *cs*OTUs from the Firmicutes in organically managed plots (Additional file 1: Fig. S7) was congruent with a separation by management type in CAP analysis of soil and root communities. The association of Firmicutes OTUs to organic plots that receive manure fertilizer was found earlier [36]. In our case, we noted that OTUs representing four families within the Firmicutes, *Peptostreptococcaceae* (*bOTU23*, *bOTU119*, genus not assigned), *Clostridiaceae* (*bOTU341*, genus *Clostridium*), *Erysipelotrichaceae* (*bOTU36*, genus *Turicibacter*), and *Lachnospiraceae* (*bOTU1403*, genus *Butyrivibrio*) had higher abundances in soil and root samples from organically managed plots (Additional file 1: Figs. S9 and S11). It is possible that the higher abundance of these OTUs is a direct result of manure application, as bacteria from these families have previously been isolated from cattle manure [59] or reported in such community surveys [60] and are also common in waste products of other livestock [61].

Although inference of ecological function from OTU data must be interpreted cautiously, we inspected the *cs*OTUs for taxa with known functions of potential importance in agriculture. Notably in soil fungi, we found two OTUs from the genus *Gibberella* (*fOTU57*, *fOTU32,* family *Nectriaceae*) that were responsive to tillage intensities and had higher abundances in no and reduced tillage samples (Additional file 1: Fig. S10). *Gibberella*, specifically *Gibberella zeae* (*fOTU57*), is a teleomorph of *Fusarium graminearum*. This pathogen of wheat causes *Fusarium* head blight disease, which is responsible for wheat yield losses worldwide [62]. Similarly, in root fungi, we noted an *Alternaria* OTU (*fOTU63* family *Pleosporaceae*) with a higher abundance in C-NT samples (Additional file 1: Fig. S12). Species of this genus are also known pathogens of wheat and cause leaf blight disease [63]. These examples could suggest that less intensive tillage systems may favor potentially pathogenic taxa. In a study examining the functional role of plant-beneficial Pseudomonads and soil suppressiveness at the FAST experiment, the soil from the O-RT plots tended to be more suppressive to the soil-borne pathogen *Pythium ultimum* to than the soil from C-NT plots (*personal communication,* Dr. M. Maurhofer, ETH Zurich). It is generally difficult to infer ecological function of a microbe solely based upon a taxonomy assignment [64]. Thus, hypotheses about microbial functions of *cs*OTUs need to be tested using other methods such as (meta-)genome or (meta-)transcriptome sequencing or by functional assays with isolated strains to experimentally test how the cropping sensitive microbes affect plant performance [65].

### Cropping system effects on microbial co-occurrence

In both soil and root meta-networks, we identified modules containing high proportions of OTUs responding similarly to different cropping practices (Fig. 4, Additional file 1: Fig. S14). We observed that *cs*OTUs grouped in distinct modules that reflected the different cropping systems. We concluded that larger groups of microbes responded in a similar manner to the specific cropping practices and therefore, clustered together in the soil and root microbial networks. The soil *cs*OTUs exhibited low to medium degrees of co-occurrence in the soil network (Fig. 5), revealing that cropping practices did not affect the highly co-occurring soil microbes, which possibly belong to “core microbiome” members [66]. This observation suggests that only the “accessory soil microbiome” could be manipulated through cropping practices. In contrast, the *cs*OTUs in the root microbiome—in particular the ones that were sensitive to organic farming—included members with high degrees of co-occurrence (see keystones below). This possibly means that influential community members can also be manipulated with organic cropping practices in the root microbiome. We see additional support for this hypothesis in the observation that *cs*OTUs also included abundant microbiome members.

Keystone taxa are thought to frequently interact with many other taxa, thereby playing an important role in the overall community [67, 68]. We found the effects of cropping system were mostly limited to non-keystone taxa despite significant effects of cropping system on β-diversity and network patterns (Figs. 2 and 4). Nevertheless, we found five keystone OTUs to be cropping sensitive in the root bacteria (Additional file 1: Table S5). Three of these—*bOTU23* and *bOTU119* (both *Peptostreptococcaceae*) and *bOTU36* (*Erysipelotrichaceae*)—are common bacteria in cattle manure or livestock waste samples [59–61], and they had higher abundances in organically managed plots (Additional file 1: Fig. S11). This finding suggests the hypothesis that manure application to soil may introduce taxa to the root microbiome with keystone function. Hence, the possible introduction of microbes from manure and their particular influence on root microbiome functioning presents a high research priority.

It is important to stress that co-occurrence networks visualize correlative relationships between taxa that include true ecological interactions (e.g., mutualism), but also non-random processes (e.g., niche-overlap), and therefore, do not necessarily reflect direct interactions between taxa [33, 69]. Future experiments will assess whether the identified keystone or cropping sensitive species directly influence other members of the microbiome or indirectly influence host performance and fitness, thereby affecting other community members [37]. Nevertheless, co-occurrence networks are a useful tool for exploring abundance patterns in complex microbial communities and could be useful in designing future experiments. For example, in combination with reference stocks of microbial isolates, plant growth experiments can be conducted in which the presence/absence or relative abundance of keystone taxa identified by network analysis can be manipulated and the effects on plant growth and development can be scored [65].

## Conclusions

The concept of “smart farming” postulates the use of state-of-the-art (originally sensing) technology to improve the quality, quantity, and sustainability of agricultural production [70]. Its central promises are targeted and site-specific interventions with “intelligent” agricultural management. Here, we propose that agricultural microbiota manipulations and management strategies shall also be considered as “smart farming.” The goal is to integrate beneficial plant microbiome traits (e.g., those improving plant growth, nutrient use efficiency, abiotic stress tolerance, and disease resistance) into sustainable agricultural production [71].

As a basis for implementing microbiota management strategies into *smart* cropping systems, we showed here to which extent and how the different cropping practices permit the manipulation of soil and root microbiota. The types of land management and tillage intensities had marked influence on dominant or well-connected bacteria and fungi in both soil and roots. Follow-up studies now need to identify the beneficial traits of cropping sensitive microbes in order to define the microbiome functions that can be manipulated through cropping practices.

## Methods

### The FAST experiment

All samples in this study were collected from the Farming Systems and Tillage (FAST) experiment near Zürich, Switzerland (47° 26′ 20″ N 8° 31′ 40″ E). For a detailed description of the FAST experiment see Wittwer et al. [18]. Briefly, the FAST experiment comprises two replicates established beside each other on the same field. The first replicate started in summer 2009 (FAST I) and the second in summer 2010 (FAST II), following a staggered start design. The FAST experiment was designed to compare conventional (C) and organic (O) managements coupled with different tillage regimes. The FAST experiment compares the four main cropping systems C-IT, C-NT, O-IT, and O-RT. Conventional plots receive synthetic mineral fertilizers, post-emergence herbicides and pesticides and are subjected to either intensive tillage (IT) or no-tillage (NT, with additional use of glyphosate). The corresponding cropping systems are referred to as conventional with intensive tillage (C-IT) or conventional without tillage (C-NT). Organically managed plots are fertilized with cattle slurry, did not receive synthetic herbicides or pesticides, and are subjected to either intensive tillage (IT) or reduced tillage (RT). The cropping systems are referred to as organic with intensive tillage (O-IT) and organic with reduced tillage (O-RT). A full-factorial design would formally require an “O-NT” treatment instead of an “O-RT” treatment. While scientifically sound, a no-till regime under organic management is not agronomically practical because of insufficient weed control without reduced tillage. Additionally, the FAST experiments comprises four cover crop treatments that are applied at the subplot level; however, for this study we only collected root and soil samples from the cover crop treatment planted with a legume species (e.g., Vicia sp.).

### Sample collection and DNA extraction

Soil and root samples from *Triticum aestivum* were collected at flowering stage in June 2014 from the second experimental replication (FAST II; Additional file 1: Fig. S1). The FAST experiment was cropped with the same winter wheat variety (cv. Titlis) but differed in seed coating between organic (untreated) and conventional (against seed-borne pathogens) systems (details: [18]). In total, 32 samples were collected (4 cropping systems (C-IT, C-NT, O-IT, O-RT) * 4 replicates * 2 sample types (soil and root)). Five soil cores (at 10–20 cm depth) were collected in each plot between wheat rows, pooled and immediately frozen at − 80 °C until DNA extraction. Additional bulk soil was collected for chemical analysis (see Additional file 1: Supplementary Methods). In each sampled subplot, whole root systems corresponding to a rooting depth of ~ 10 cm were collected from five plants and pooled. The roots were rinsed with tap water to remove soil debris, dried by blotting with sterile paper, and stored at − 80 °C until DNA extraction. Our sampling method does not discriminate between microbes inhabiting the inner root tissue and the root surface and for simplicity; we refer to these combined habitats of root-associated microbes as “root” samples.

The entire root systems were first lyophilized for 48 h and then ground to a fine powder in a ball mill. DNA was extracted from a 300-mg soil or root (dry weight) subsample using the NucleoSpin Soil DNA extraction kit (Machery-Nagel GmbH & Co. KG, Düren, Germany) according to the manufacturer’s instructions, except each sample was extracted twice and the supernatants pooled to maximize DNA yield. Extracted DNA was quantified using a Quant-iT Picogreen dsDNA Assay Kit (Invitrogen, Eugene, OR, USA) on a Varian Cary Eclipse fluorescence spectrometer (Agilent Technologies, Santa Clara, CA, USA).

### PCR, library preparation, and sequencing

The 16S rRNA gene amplicon library was generated using the PCR primers 799F [72] and 1193R [73]. The ITS amplicon library was generated using the PCR primers fITS7 [74] and ITS4 [75]. The primers were extended at the 5′end with an error-tolerant barcode for multiplexed library sequencing (Additional file 2). We refer to Additional file 1: Supplementary Methods for details in PCR setup, cycling conditions (Additional file 1: Table S1) and the protocol for library preparation. The libraries were sequenced on the MiSeq Instrument (Illumina, San Diego, USA) using a 600-cycle v3 Sequencing kit, paired-end 2 × 300 cycle sequencing mode at the Functional Genomics Center Zurich (www.fgcz.ch).

### Bioinformatics

Raw reads were processed using a custom-developed bioinformatics pipeline whose command-line based script is provided as Additional file 4. Reads were pre-quality filtered and trimmed at the 3′-end to 280 bp using PRINSEQ [76] and then merged with FLASH [77]. Sequences were de-multiplexed using Cutadapt [78] and were quality-filtered with PRINSEQ. For operational taxonomic unit (OTU) delineation the 16S rRNA gene sequences were trimmed to the fixed length of 360 bp, sorted by abundance, de-replicated, and clustered to OTUs (≥ 97%, singletons removed) with UPARSE [79]. Chimeric sequences were screened using UCHIME [80] against the GOLD database [81] and removed. Taxonomy assignment was performed using the SILVA database (v119; [82] with the RDP classifier as implemented in QIIME [83]. ITS sequences were processed similarly, except they were trimmed to 220 bp and chimeric sequences were screened against the UNITE database [84]. Taxonomy was assigned using the UNITE database (v7.0) with the RDP classifier in QIIME.

### Data analysis in R

All statistical analyses were conducted in R v3.3.0 [85]. The R script and all necessary input files are provided as Additional file 5. Additionally, a workflow of the data analysis steps presented below and the figures generated from each step is given in Additional file 1: Fig. S2. Briefly, the bacteria OTUs (bOTUs) and taxonomy tables were filtered to exclude OTUs classified as chloroplasts and mitochondria. Similarly, fungi OTUs (fOTUs) classified as plant, protist, or whose kingdom or phylum was unassigned were removed.

#### Alpha diversity

Rarefaction analysis was performed in QIIME on the filtered OTU tables that were exported from R. The OTU tables were rarefied from 1000 to 37,000 (bacteria) or 1000 to 48,000 (fungi) sequences per sample with a step size of 1000 and 100 iterations at each step. Estimates of α-diversity (observed OTU richness) were calculated at each rarefaction level in QIIME (Additional file 1: Fig. S3a). We tested the effects of sample type and cropping system on observed species richness for each kingdom individually. For this, we randomly selected one file containing α-diversity estimations at 11,000 (bacteria) and 9000 (fungi) sequences per sample from QIIME. We tested for differences between soil and root sample using Student’s *t* test. We then assessed the effects of experimental block and cropping system on observed species richness using two-way ANOVA within each kingdom and sample type separately. Because cropping system was confounded within experimental block, we did not test for the Block*Cropping System interaction. Significant differences between cropping systems were assessed using Tukey’s Honest Significant Differences test using the R package TukeyC [86].

#### Beta diversity

We conducted a general analysis of β-diversity on the bacterial and fungal communities comparing soil and root samples together (Additional file 1: Fig. S3a) and then subsequently we performed more specific hypothesis testing on the soil and root communities individually (Additional file 1: Fig. S3b). For the general analysis, we normalized the filtered OTU sequence counts for each microbial kingdom separately using the “trimmed means of M” (TMM) method with the BioConductor package *edgeR* (10) and expressed the normalized counts as relative abundance counts per million (CPM). We then performed unconstrained principle coordinates analysis (PCoA) on Bray-Curtis dissimilarities to quantify the major variance components of β-diversity in each kingdom. Ordination analyses were performed using the R package *phyloseq* [87]. We tested for sample type and cropping system effects on community dissimilarity with permutational analysis of variance (PERMANOVA) using the functions *adonis* in the *vegan* package with 10^4^ permutations [88].

For the separate in-depth analyses of each microbial kingdom and for each sample type (soil and root), we additionally applied the following sequence count threshold to the OTU tables: we selected OTUs with at least two sequences (avoiding single-count OTUs) in at least four samples (the number of replicates per treatment). We considered OTUs remaining after this thresholding step to be the soil and root communities. We normalized the communities using the TMM method and expressed the values as relative abundance CPM. We then performed multivariate analysis of microbial diversity based on the steps outlined by Anderson and Willis [89]. This included a constrained analysis of principal coordinates (CAP) testing the effect of the cropping systems, statistical testing of the cropping system hypothesis, and identification of the OTUs responsible for the observed effects (see below). All ordination analyses were performed using the R package *phyloseq* [87]. Statistical significance of the CAP was assessed using the *permutest* function in the *vegan* package [88] with 10^4^ permutations. We tested for cropping system effects on community dissimilarity with permutational analysis of variance (PERMANOVA) and permutational analysis of multivariate dispersions (BETADISP) using the functions *adonis* and *betadisp*, respectively, in the *vegan* package with 10^4^ permutations. Where applicable, pairwise differences between the cropping systems were assessed with the function *pairwise.perm.manova* from the package *RVAideMemoire* [90]*.*

#### Identification of cropping sensitive OTUs (*cs*OTUs)

We employed complementary approaches to identify the OTUs responsible for the observed effects. We used correlation based indicator species analysis with the R package *indicspecies* [91] to calculate the point-biserial correlation coefficient (*r*) of an OTU’s positive association to one or a combination of cropping systems. The analysis was conducted with 10^4^ permutations and considered significant at *p* < 0.05. Additionally, we tested for differential OTU abundance between one or more of the cropping systems of soil and root communities (same thresholded OTU tables) of both kingdoms using likelihood ratio tests (LRT) with the R package *edgeR* [41]. OTUs whose abundances were identified as differing between one or more of the cropping systems at a false discovery rate (FDR) corrected value of *p* < 0.05 were considered to be cropping system responsive. We then defined OTUs that were confirmed by both indicator species analysis *and* LRT as *c*ropping *s*ensitive OTUs (*cs*OTUs).

#### Bipartite networks

We visualized the significant (*p* < 0.05) OTU associations to one or more of the different cropping system from the indicator species analysis using bipartite networks. The networks were constructed using the Fruchterman-Reingold layout with 10^4^ permutations as implemented in the R package *igraph* [92].

#### Co-occurrence networks

We constructed two types of co-occurrence networks. For all networks, we utilized the TMM normalized CPM counts and conducted Spearman rank correlations between OTUs and visualized the positive, significant correlations (*ρ* > 0.7 and *p* < 0.001). All networks were visualized with the Fruchterman-Reingold layout with 10^4^ permutations in *igraph*.

For the in-depth assessment of soil and root bacterial and fungal communities, we performed Spearman rank correlations between all pairs of bacteria and all pairs of fungi OTUs within the soil and root communities separately. We calculated the descriptive and topological network properties with *igraph.* These included: the total number of network nodes (representing OTUs), total number of edges (connections between nodes representing positive, significant correlations between OTUs), and degrees of co-occurrence (number of direct correlations to a node).

We then constructed meta-networks to visualize correlations between bacteria and fungi in the soil and root communities. For this, we combined the TMM normalized CPM counts of bacteria and fungi into separate OTU tables for the soil and root communities. We performed Spearman rank correlations between all pairs of bOTUs and fOTUs. We calculated the network properties mentioned above, and additionally, to explore community structure within the soil and root meta-networks, we identified network modules. These are substructures of nodes with a higher density of edges within groups than between them. For this we utilized the *greedy optimization of modularity* algorithm [93] as implemented in *igraph*.

Microbial taxa that frequently co-occur with other taxa in microbial co-occurrence networks are thought to be ecologically important and potentially play a key role within the microbiome [37, 38]. We identified keystone OTUs separately for the soil and root meta-networks and defined them as those nodes within the top 1% of node degree values of each network. We prioritized this simple definition over a more complex method (e.g., based on high degree and low betweenness centrality) because both definitions uncovered largely the same sets of keystone OTUs (data not shown).

## Additional files

Additional file 1: A PDF containing supplementary methods, results, discussion, references, figures and tables. The Supplementary Methods contain the details about the chemical soil analysis, PCR setup, library preparation and sequencing. The Supplementary Results comprise the global taxonomic profiles of soil and root bacterial and fungal communities and the taxonomic patterns of *cs*OTUs. We discuss the cropping system effects on soil microbial communities and on microbial α-diversity in the Supplementary Discussion. Supplementary Figures: Figure S1. - Experimental layout of the FAST experiment. Figure S2. - Graphical overview of data analysis. Figure S3. - Taxonomic profiles at phylum level. Figure S4. - Unconstrained PCoA ordinations. Figure S5. - Rarefaction curves. Figure S6. - Defining cropping sensitive bacteria and fungi in soil and root samples. Figure S7. and Figure S8. - Mean relative abundances of cropping sensitive OTUs at phylum and OTU level, respectively. Figure S9. - Abundant cropping sensitive bacteria bOTUs in soil. Figure S10. - Abundant cropping sensitive fungi fOTUs in soil. Figure S11. - Abundant cropping sensitive bacteria bOTUs in roots. Figure S12. - Abundant cropping sensitive fungi fOTUs in roots. Figure S13. - Separate co-occurrence networks of bacteria and fungi in soil and root samples. Figure S14. - Defining modules in root and soil networks. Supplementary Tables: Table S1. - PCR cycling conditions. Table S2. - PERMANOVA results for testing the effects of *Block*, *Sample type* and *Cropping System*. **Table S3.** - Statistic results testing for differences in α-diversity. **Table S4.** - PERMANOVA results testing the effects of *Block* and *Cropping System* on bacterial and fungal communities in soil and root samples. **Table S5.** - Characteristics of keystone OTUs. (PDF 2862 kb)

Additional file 2: An XLSX table contains the experimental design (Sample ID, sample type and cropping system), chemical soil data and sequencing information (barcodes and sequence counts). (XLSX 46 kb)

Additional file 3: An XLSX table reporting the indicator species and edgeR results and the assignments to cropping sensitive OTUs and network modules. This information is provided in separate sheets for the bacteria and fungi in soil and roots. (XLSX 278 kb)

Additional file 4: A zip archive comprising the command line code and necessary input files needed to replicate bioinformatic analysis. (ZIP 628 kb)

Additional file 5: A zip archive with the R script and necessary input files needed to reproduce all statistical analyses and graphics. (R 159 kb)
