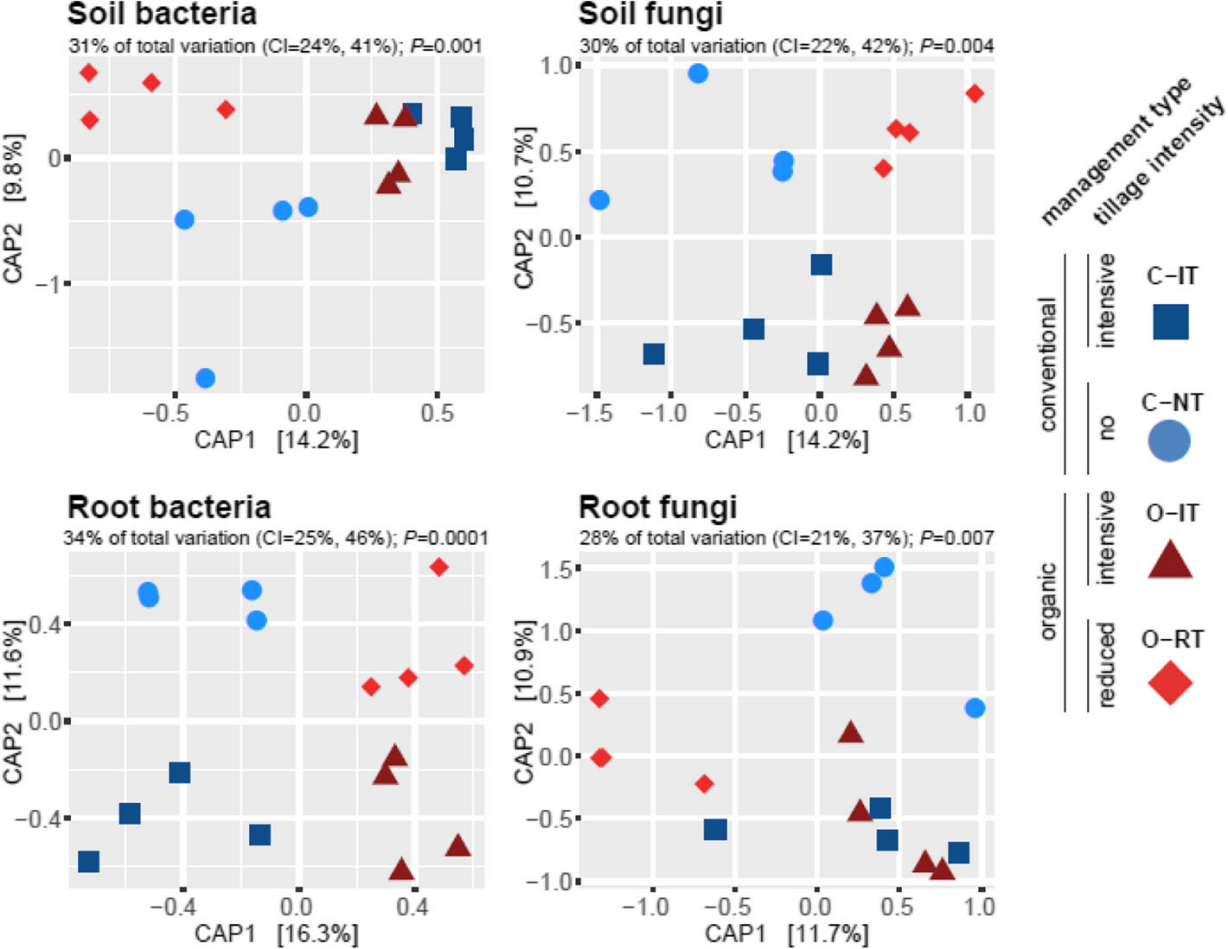# Correction to: Cropping practices manipulate abundance patterns of root and soil microbiome members paving the way to smart farming

**DOI:** 10.1186/s40168-018-0456-x

**Published:** 2018-04-24

**Authors:** Kyle Hartman, Marcel G. A. van der Heijden, Raphaël A. Wittwer, Samiran Banerjee, Jean-Claude Walser, Klaus Schlaeppi

**Affiliations:** 1 Plant-Soil Interactions, Department of Agroecology and Environment, Agroscope, Zurich, Switzerland; 2 Institute for Evolutionary Biology and Environmental Studies, University of Zurich, Zurich, Switzerland; 3 Genetic Diversity Centre, ETH Zurich, Zurich, Switzerland; 4 Plant-Microbe Interactions, Institute of Environmental Biology, Faculty of Science, Utrecht University, Utrecht, The Netherlands

## Correction

Following publication of the original article [1], the authors reported that while the ordination graphs are all correct, the symbols in the legend are wrong. During revisions the authors moved the legend from the bottom to the right side of the graphs, and thereby a mistake with symbol assignment occurred. The corrected Figure is given below: