# Correction to: Cropping practices manipulate abundance patterns of root and soil microbiome members paving the way to smart farming

**DOI:** 10.1186/s40168-020-00855-4

**Published:** 2020-05-17

**Authors:** Kyle Hartman, Marcel G. A. van der Heijden, Raphaël A. Wittwer, Samiran Banerjee, Jean-Claude Walser, Klaus Schlaeppi

**Affiliations:** 1 Plant-Soil Interactions, Department of Agroecology and Environment, Agroscope, Zurich, Switzerland; 2 Institute for Evolutionary Biology and Environmental Studies, University of Zurich, Zurich, Switzerland; 3 Genetic Diversity Centre, ETH Zurich, Zurich, Switzerland; 4 Plant-Microbe Interactions, Institute of Environmental Biology, Faculty of Science, Utrecht University, Utrecht, The Netherlands

**Correction to: Microbiome 6, 14 (2018)**

**https://doi.org/10.1186/s40168-017-0389-9**

Following publication of the original article [1], the authors reported an error in the Additional files 4 and 5. The correct Additional files have been included in this correction.

## Supplementary information

**Additional file 4.** A zip archive comprising the command line code and necessary input files needed to replicate bioinformatic analysis.

**Additional file 5.** A zip archive with the R script and necessary input files needed to reproduce all statistical analyses and graphics.
