# Supplementary material for: Cropping practices manipulate abundance patterns of root and soil microbiome members paving the way to smart farming
Source: Microbiome. 2018 Jan 16;6:14. doi: 10.1186/s40168-017-0389-9 (PMC5771023; doi:10.1186/s40168-017-0389-9)
Supplement: Supplementary file 1 — A PDF containing supplementary methods, results, discussion, references, figures and tables. The Supplementary Methods contain the details about the chemical soil analysis, PCR setup, library preparation and sequencing. The Supplementary Results comprise the global taxonomic profiles of soil and root bacterial and fungal communities and the taxonomic patterns of csOTUs. We discuss the cropping system effects on soil microbial communities and on microbial α-diversity in the Supplementary Discussion. Supplementary Figures: Figure S1. - Experimental layout of the FAST experiment. Figure S2. - Graphical overview of data analysis. Figure S3. - Taxonomic profiles at phylum level. Figure S4. - Unconstrained PCoA ordinations. Figure S5. - Rarefaction curves. Figure S6. - Defining cropping sensitive bacteria and fungi in soil and root samples. Figure S7. and Figure S8. - Mean relative abundances of cropping sensitive OTUs at phylum and OTU level, respectively. Figure S9. - Abundant cropping sensitive bacteria bOTUs in soil. Figure S10. - Abundant cropping sensitive fungi fOTUs in soil. Figure S11. - Abundant cropping sensitive bacteria bOTUs in roots. Figure S12. - Abundant cropping sensitive fungi fOTUs in roots. Figure S13. - Separate co-occurrence networks of bacteria and fungi in soil and root samples. Figure S14. - Defining modules in root and soil networks. Supplementary Tables: Table S1. - PCR cycling conditions. Table S2. - PERMANOVA results for testing the effects of Block, Sample type and Cropping System. Table S3. - Statistic results testing for differences in α-diversity. Table S4. - PERMANOVA results testing the effects of Block and Cropping System on bacterial and fungal communities in soil and root samples. Table S5. - Characteristics of keystone OTUs. (PDF 2862 kb) [file 40168_2017_389_MOESM1_ESM.pdf]

Additional file 1

Cropping Practices Manipulate Abundance Patterns Of Root And Soil Microbiome Members Paving The Way To Smart Farming

Kyle Hartman[1,2], Marcel G.A. van der Heijden[1,2,4], Raphaël A. Wittwer[1], Samiran Banerjee[1], Jean-Claude Walser[3], and Klaus Schlaeppi[1*]

SUPPLEMENTARY METHODS

Chemical soil analysis

We collected soil samples for chemical analysis in March 2014, before the application of fertilizers or weed control measures. We took 20 soil cores with a soil auger (ø 2.5 cm) in the inner 2 x 10 m of each subplot to a soil depth of 0-20 cm and combined them to one meta-sample per plot. The samples were then sieved at 2 mm and kept at 4ºC until analysis. Soil samples were analyzed for pH, organic and total C, total N, and soil texture, extracted with 1:10 ammonia-acetate-EDTA and determined according to the reference methods of the Swiss Federal Research Stations [1].

16S PCR and library preparation

The 16S amplicon library was generated using the PCR primers 799F [2] and 1193R [3]. The primers were extended at the 5`end with an error-tolerant barcode for multiplexed library sequencing (**Supplementary Data S1**). PCR reactions were performed on a iCycler instrument (BioRad, Hercules, CA, USA) using the 5PRIME Hot Master Mix PCR system (5 PRIME, Gaithersburg, MD USA) with the cycling conditions in **Table S1**. Each 20 μL reaction contained: 8 μL 5PRIME Hot Master Mix, 0.3 % BSA, 200 nM each primer, and 2 ng and 10 ng of DNA template for soil and root reactions respectively, and the remaining volume sterile distilled water. PCR reactions were conducted in quadruplicates and pooled together before inspecting 3 μL of each sample on a 1 % agarose gel at 90 V for 45 min for correct size and absence of contamination in non-template reactions. PCR reactions were then purified using the NucleoSpin Gel and PCR Clean up Kit (Machery-Nagel GmbH & Co. KG, Düren, Germany) according to the manufacturer's instructions. The purified reactions were quantified using the same Picogreen assay described above and pooled in equal amounts (100 ng / sample), after which the library volume was reduced using a CentriVap centrifugal vacuum concentrator (Labconco Corp., Kansas City, MO, USA). The concentrated library was mixed with loading dye, split equally between 2 lanes of a 1.2 % agarose gel to separate the 'bacteria band' from the ~800 bp mitochondria product also produced by the primers. Bacteria bands were cut and these gel fragments purified using the kit

described above, eluted in 50 μL of the supplied elution buffer and measured using a Qubit assay (Agilent Technologies, Santa Clara, USA).

ITS PCR and library preparation

The ITS amplicon library was generated using the PCR primers fITS7 [4] and ITS4 [5]. The primers were extended at the 5`end with an error-tolerant barcode for multiplexed library sequencing (**Supplementary Data S1**). PCR reactions were performed on an iCycler instrument (BioRad, Hercules, CA, USA) using the DreamTaq PCR system (Thermo-Fisher Scientific, Waltham, MA, USA) with the cycling conditions in **Table S1**. Each 20 μL reaction contained: 10 μL DreamTaq PCR MasterMix (DreamTaq DNA Polymerase, 1x DreamTaq Buffer, 2 mM $MgCl_2^+$, 200 μM each dNTP), supplemental $MgCl_2^+$ to 2.75 mM, 0.3 % BSA, 500 nM of the forward primer, 200 nM of the reverse primer, 10 ng of DNA template for both soil and root samples, and the remaining volume sterile distilled water. PCR reactions were conducted in quadruplicates and pooled together before validation by gel electrophoresis. The reactions were quantified using a Picogreen assay and pooled in equal amounts (200 ng / sample). The volume of the pooled library was reduced using a CentriVap centrifugal vacuum concentrator (Labconco Corp., Kansas City, MO, USA), mixed with loading dye and subjected to separation on a 1.5% agarose gel. The bands between 300-500 bp were cut from the gel and purified with the NucleoSpin Gel and PCR Clean up Kit (Machery-Nagel GmbH & Co. KG, Düren, Germany) according to the manufacturer's instructions, eluted in 50 μL of the supplied elution buffer, and the DNA quantified using a Qubit assay (Agilent Technologies, Santa Clara, USA).

Library sequencing

Preparation of the 16S and ITS amplicon libraries was conducted as follows: The TruSeq DNA Sample Prep Kit v2 (Illumina, San Diego, CA, USA) was used following the manufacturer's instructions. Briefly, the amplicon samples were end-repaired and polyadenylated. TruSeq adapters containing the index for multiplexing were ligated to the amplicon samples. The ligated samples were run on a 2% agarose gel and the desired fragment length was excised (50 bp +/- the target fragment length). DNA from the gel was purified with MinElute Gel Extraction Kit (Qiagen, Hilden, Germany). Fragments containing TruSeq adapters on both ends were selectively enriched with PCR using 4 cycles. The quality and quantity of the enriched libraries were validated using Qubit and Tapestation (Agilent Technologies, Santa Clara, CA USA). The libraries were normalized to 4 nM in Tris-Cl 10 mM, pH 8.5 with 0.1% Tween 20. The library was sequenced on the Illumina MiSeq Personal Sequencer (Illumina, San Diego, USA) using a 600 cycle v3 Sequencing kit (Cat n° MS-102-3003), paired-end 2x 300 cycle sequencing mode at the Functional Genomics Center Zurich (www.fgcz.ch).

SUPPLEMENTARY RESULTS

Taxonomic profiles of soil and root bacterial and fungal communities

Differences between the soil and root microbiota were evident in the taxonomic profiles of both sample types. We noted 30 bacteria phyla present in soil samples, with Proteobacteria (39.3%), Actinobacteria (31.2%), and Firmicutes (6.3%) having the highest relative abundances (**Fig. S3**). We found 25 different phyla in root samples with Actinobacteria (41%), Proteobacteria (39.7%), and Bacteroidetes (10.7%) being most abundant. In fungal communities, soils contained at least six phyla, with abundant Ascomycota (72.2%), Basidiomycota (9.4%) and Zygomycota (4.9%). OTUs from unassigned phyla made up ~11.9% of fungi OTUs. In root samples, we also found OTUs from at least six phyla with abundant Ascomycota (80.6%) and Basidiomycota (16.2%). OTUs from unassigned phyla comprised ~3% of the community. We found that the proportion of sequences from the phylum Glomeromycota, which contain the arbuscular mycorrhizal fungi (AMF), was generally very low in both sample types (mean relative abundance of 1% in soil samples and 0.02% in root samples), confirming that the primer combination fITS7 - ITS4 is suboptimal to characterize AMF communities (**Fig. S3**). It is known that general fungal ITS primers poorly resolve and discriminate AMF taxa [6,7].

Taxonomic patterns of csOTUs

*C*ropping *s*ensitive OTUs (*cs*OTUs) were identified based on indicator species analysis and using likelihood ratio tests. The 53 *cs*OTUs in the soil bacterial community (**Fig. S6**) comprised at least 11 phyla, with the majority of community sequences belonging to the Actinobacteria (25.4%), Proteobacteria (22.5%) and Firmicutes (18.4%). We noted that specific phyla tended to respond to specific management systems and tillage regimes. OTUs belonging to the Firmicutes favored organically managed plots (**Fig. S7**). Bacteroidetes OTUs tended towards higher mean abundances in no-till and reduced tillage treatments; whereas mean abundances of OTUs from the Acidobacteria and Verrucomicrobia were higher in the full-tillage treatments. OTUs from the Chloroflexi tended to favor the O-RT system. We also examined the taxonomic assignment and mean relative abundances of the individual *cs*OTUs across the four cropping systems (**Fig. S8**). We noted higher relative abundances of Firmicutes OTUs *bOTU36* (family *Erysipelotrichaceae*), *bOTU23, bOTU119* (both *Peptostreptococcaceae*) and *bOTU341* (*Clostridiaceae*) in organically managed plots, consistent with patterns seen at the phylum level (**Figs. S7-S9**). We observed similar patterns for OTUs from the phylum Acidobacteria (*bOTU806*, *bOTU885*, *bOTU238*, *bOTU651*, family unassigned), which were consistently more abundant in plots receiving intensive tillage (**Figs. S8, S9**).

The bulk soil fungal community comprised 70 *cs*OTUs (**Fig. S6**) classified into at least six different phyla, with Ascomycota (81.2% of sequences) unassigned (8.5%) and Basidiomycota (5.5%) being the most abundant (**Fig. S7**). We observed a number of known Ascomycota OTUs, possibly belonging to pathogenic fungi, that were abundant in C-NT system (*fOTU57*, family *Nectriaceae*) and organically managed plots (*fOTU32 Nectriaceae; fOTU25 and fOTU1628 Sporormiaceae*) (**Figs. S8, S10**). We also noted

that a single OTU from the phylum Glomeromycota (*fOTU980*, family *Diversisporaceae*) was absent in C-IT samples and enriched in O-IT.

In root bacterial communities, the 63 *cs*OTUs (**Fig. S6**) were classified into ten different phyla, with Actinobacteria, Proteobacteria and Firmicutes having the highest relative abundances (73.4%, 13,2%, and 9.5% of sequences, respectively; **Fig. S7**). Across the four cropping systems, OTUs from the Actinobacteria were equally well represented. OTUs from the Proteobacteria and Bacteroidetes, were more abundant in reduced and no-tillage plots. Like in the soil bacterial community, the Firmicutes were generally more abundant in root samples from organically managed plots. This appeared to be driven by the increased abundance of several OTUs from the family *Peptostreptococcaceae* (*bOTU23, bOTU119), Clostridiaceae* (*bOTU341*), *Erysipelotrichaceae* (*bOTU36*), and *Lachnospiraceae* (*bOTU1403*), a family that was exclusive to organically managed plots. (**Figs. S8, S11**).

The 36 *cs*OTUs (**Fig. S6**) in root fungal communities were classified into at least three phyla. Most sequences belonged to the Ascomycota (75.9%), followed by unassigned phyla (14.1%), and Basidiomycota (9.8%; **Fig. S7**). We noted that OTUs from the Ascomycota favored the C-NT system and, to a lesser extent, the organically managed plots. The O-RT system supported a higher abundance of OTUs belonging to unassigned phyla and the Basidomycota. Many of the cropping sensitive OTUs were unassigned at lower taxonomic levels (**Fig. S8**). However, in the Ascomycota, *fOTU63* (*Pleosporaceae)* and *fOTU97* (*Phaeosphaeriaceae)* were abundant in the C-NT system, while the *Psathyrellaceae fOTU86* was abundant in the O-RT system (**Figs. S8, S12**). We also noted a number of OTUs from the family *Lasiosphaeriaceae* with higher mean abundances in the O-IT treatment.

SUPPLEMENTARY DISCUSSION

Cropping system effects on soil microbial communities

We found significant effects of cropping system on soil microbial communities, explaining approximately 30% of the total variation in both bacteria and fungi (**Fig. 2**). More specifically, bacterial communities were more strongly separated by the different tillage regimes rather than by management type, with the biggest differences between intensive tillage samples and those receiving less intensive tillage (**Table S4**). This finding is somewhat unexpected given that earlier work has shown that the addition of manure, as is the case in the organically managed plots, can result in substantial shifts in soil bacterial community [8–11]. Moreover, bacteria are generally thought to be relatively unaffected by tillage practices, given their small cell size and constrained dispersal and are therefore, less likely to be affected by the homogenization of soil microsites [12,13].

It has also been suggested that bacteria introduced into soils from manure amendments do not become prominent [9] and that any bacterial community compositional shifts as a result of manure additions tend to diminish over time [8–10]. However, these results would seemingly conflict with a number of recent studies that have profiled microbial communities in soils receiving inorganic and organic fertilizer and found substantial differences between the two fertilizer regimes [14–17]. For example, Hartmann et al., [15] profiled soil microbial communities from a long-term (>20 years) Swiss agricultural experiment comparing five different management systems receiving either mineral fertilizer or farm yard manure. They found that the application of farm yard manure was the primary driving force behind bacterial community dissimilarity. Thus, we hypothesize our findings could be attributed to two reasons. First, because we collected soil samples over two months after the final application of manure in the organically managed plots, any initial changes to the bacterial community may have largely disappeared by the time the samples were collected. Second, the abovementioned studies reporting manure induced shifts in bacterial community composition were all conducted on long-term agricultural trials under decades of manure amendment. Although the entire FAST experimental site has been under organic management since 2002, the cropping treatments were only established for FAST II in 2010 [18]. Therefore, our results may be indicative of the relatively short period of manure amendments at the site.

In contrast to soil bacteria, constrained ordinations of soil fungal communities revealed that differences between conventional and organic management types explained most of the variation (**Fig. 2**). Despite the relatively short term management of the FAST site, our results are more in accordance with previous studies on long-term (>20 years) agricultural trials that reported significant effects of organic management with manure fertilization on soil fungal community composition [15,19]. Studies on soil communities subjected to organic management with manure additions over the short term (typically less than 10 years) have tended to report no significant differences in fungal community structure between manure amended and non-amended soils [20,21]. However, these shorter-term studies relied on older molecular tools, which may be less precise in capturing subtle community shifts compared to amplicon sequencing [19].

Nevertheless, there is evidence that the addition of manure to soils represents an input of external microbes that could affect strong changes in the diversity and composition of both bacterial and fungal communities over the course of a growing season [19,22]. With this in mind, our results highlight the need for future studies to assess the temporal variability in soil communities receiving external microbial inputs, such as manure. Sampling at multiple time points, including before manure application, would shed light on the dynamics of the bacterial and fungal communities during the course of the growing season. This could help to improve estimates of microbial α-diversity, which have been shown to exhibit greater temporal variability than across different land use types [23]. Furthermore, future studies would benefit from the inclusion of manure samples in high-throughput sequencing runs for the direct identification of manure-derived bacteria and fungi OTUs based on sequence similarity.

We found that an increase in tillage intensity from reduced tillage to intensive tillage resulted in significantly different soil fungal communities in organically managed plots; whereas the same was not observed between no-till and intensive tillage samples in conventional plots (**Table S4**). This suggests that tillage effects on soil fungal communities may depend on other factors, such as management type. Other previous work on the effects of soil disturbance events on soil fungi have often focused on AMF as a group of fungi sensitive to increasing tillage intensity [24–26]. However, we are unable to draw conclusions about effects of tillage on AMF communities at the FAST site due to very low abundances of AMF sequences (**Fig. S3**). It is generally thought that tillage affects soil AMF communities through physical destruction of dense hyphal networks [27]. Such mechanisms of physical disturbance are also thought to influence communities of general soil fungi, and therefore less soil disturbance and more heterogeneous resource distribution, common of no till and reduced tillage systems, may promote fungal communities [28]. Many hypotheses about the effects of tillage on fungal communities also focus on indirect effects, namely that tillage influences edaphic factors like soil organic carbon content [29,30] and soil nutrient pools like extractable P [31], which have been shown to influence soil fungal community composition. Similarly, our unconstrained ordination analyses revealed that differences in pH explained approximately 24% and 27% of community variation in the soil bacterial and fungal communities, respectively (**Fig. S4**). These results are generally consistent with previous findings showing soil pH as a significant driver of primarily bacterial community composition [32,33], but also of fungi [34]. However, it is important to stress that our findings were less the result of a true pH gradient across multiple samples and more the result of a low pH value in one subplot.

Cropping system effects on microbial α-diversity

We have assessed the effects of cropping systems on observed bacteria and fungi OTU richness in both soil and root samples, confirming that soils were more diverse than root microbial communities [35,36]. With respect to the effects of cropping system, we found the soil bacteria and fungi tended to be richest in the O-IT system (**Fig. S5, Table S3**). These observations are in accordance with previous studies reporting higher soil microbial richness in organically managed compared to conventionally managed soils (bacteria: 29, 50, 51; fungi: 29, 52). However, there are also studies reporting no

differences between conventional and organic managements [40,41]. We speculate that timing differences between application and sampling might explain conflicting results, in that any enhanced diversity effects might disappear in the time span between manure application and sampling.

The effects of differential soil managements on the root microbes appear to vary depending on the root compartment analyzed. Edwards et al., [42] found differences in bacteria α-diversity in the rhizosphere but not rhizoplane and endosphere compartments when comparing samples from conventional and organically managed cropping systems. Also Seghers et al., [43] found no difference in maize root endophyte richness (bacteria and fungi) in samples taken from conventionally and organically managed plots. Soil management seems to affect microbial communities to lesser extents the more intimate they associate with their host plant. We think that our root sampling method without physical (no sonication) or chemical (no detergent or bleach) separation from the rhizosphere compartment yields a rather low-intimacy type of compartment and we expected to find impacts by soil management. Indeed, we found effects of cropping practices on observed root OTU richness. We found significantly higher richness in in O-IT plots compared to conventionally managed plots for the bacteria (**Fig. S5; Table S3**).

Taken together, we find enhanced richness in root and soil microbiota in O-IT systems. We think that the application of animal manure as fertilizer coupled with structural disturbance presents a likely explanation for the enhanced diversity in organic intensive tillage systems.

SUPPLEMENTARY REFERENCES

1. Eidgenössische Forschungsanstalten FAL, RAC F. Schweizerische Referenzmethoden der Eidgenössischen landwirtschaftlichen Forschungsanstalten. Zürich: Eidg. Forschungsanstalt für Landwirtschaftlichen Pflanzenbau, FAP; 1996.
2. Chelius MK, Triplett EW. The Diversity of Archaea and Bacteria in Association with the Roots of Zea mays L. Microb. Ecol. 2001;41:252–63.
3. Bodenhausen N, Horton MW, Bergelson J. Bacterial communities associated with the leaves and the roots of Arabidopsis thaliana. PLoS One. 2013;8:e56329.
4. Ihrmark K, Bödeker ITM, Cruz-Martinez K, Friberg H, Kubartova A, Schenck J, et al. New primers to amplify the fungal ITS2 region--evaluation by 454-sequencing of artificial and natural communities. FEMS Microbiol. Ecol. 2012;82:666–77.
5. White T, Bruns T, Lee S, Taylor J. Amplification and direct sequencing of fungal ribosomal RNA Genes for phylogenetics. In: Innis M, Gelfland D, Sninsky J, White T, editors. PCR Protoc. A Guid. to Methods Appl. San Diego, CA: Academic Press; 1990. p. 315–22.
6. Stockinger H, Krüger M, Schüßler A. DNA barcoding of arbuscular mycorrhizal fungi. New Phytol. 2010;187:164.
7. Kohout P, Sykorova Z, Ctvrtlikkova M, Rydlova J, Suda J, Vohnik M, et al. Surprising spectra of root-associated fungi in submerged aquatic plants. FEMS Microbiol. Ecol. 2012;80:216–35.
8. Hammesfahr U, Heuer H, Manzke B, Smalla K, Thiele-Bruhn S. Impact of the antibiotic sulfadiazine and pig manure on the microbial community structure in agricultural soils. Soil Biol. Biochem. 2008;40:1583–91.
9. Heuer H, Focks A, Lamshöft M, Smalla K, Matthies M, Spiteller M. Fate of sulfadiazine administered to pigs and its quantitative effect on the dynamics of bacterial resistance genes in manure and manured soil. Soil Biol. Biochem. 2008;40:1892–900.
10. Jechalke S, Focks A, Rosendahl I, Groeneweg J, Siemens J, Heuer H, et al. Structural and functional response of the soil bacterial community to application of manure from difloxacin-treated pigs. FEMS Microbiol. Ecol. 2014;87:78–88.
11. Jangid K, Williams MA, Franzluebbers AJ, Sanderlin JS, Reeves JH, Jenkins MB, et al. Relative impacts of land-use, management intensity and fertilization upon soil microbial community structure in agricultural systems. Soil Biol. Biochem. 2008;40:2843–53.
12. Strickland MS, Rousk J. Considering fungal:bacterial dominance in soils – Methods, controls, and ecosystem implications. Soil Biol. Biochem. 2010;42:1385–95.
13. Hendrix P, Parmelee R, Crossley Jr D, Coleman D, Odum E, Groffman P. Detritus Food Webs in Conventional and No- Tillage Agroecosystems. Bioscience. 1986;36:374–80.

14. Li F, Chen L, Zhang J, Yin J, Huang S. Bacterial Community Structure after Long-term Organic and Inorganic Fertilization Reveals Important Associations between Soil Nutrients and Specific Taxa Involved in Nutrient Transformations. Front. Microbiol. 2017;8:10.3389/fmicb.2017.00187.

15. Hartmann M, Frey B, Mayer J, Mäder P, Widmer F. Distinct soil microbial diversity under long-term organic and conventional farming. ISME J. 2014;9:1177–1194.

16. Ding J, Jiang X, Ma M, Zhou B, Guan D, Zhao B, et al. Effect of 35 years inorganic fertilizer and manure amendment on structure of bacterial and archaeal communities in black soil of northeast China. Appl. Soil Ecol. 2016;105:187–95.

17. Francioli D, Schulz E, Lentendu G, Wubet T, Buscot F, Reitz T. Mineral vs. organic amendments: Microbial community structure, activity and abundance of agriculturally relevant microbes are driven by long-term fertilization strategies. Front. Microbiol. 2016;7:10.3389/fmicb.2016.01446.

18. Wittwer RA, Dorn B, Jossi W, van der Heijden MGA. Cover crops support ecological intensification of arable cropping systems. Sci. Rep. 2017;7:10.1038/srep41911.

19. Lentendu G, Wubet T, Chatzinotas A, Wilhelm C, Buscot F, Schlegel M. Effects of long-term differential fertilization on eukaryotic microbial communities in an arable soil: A multiple barcoding approach. Mol. Ecol. 2014;23:3341–55.

20. Marschner P, Kandeler E, Marschner B. Structure and function of the soil microbial community in a long-term fertilizer experiment. Soil Biol. Biochem. 2003;35:453–61.

21. Lejon DPH, Sebastia J, Lamy I, Chaussod R, Ranjard L. Relationships between soil organic status and microbial community density and genetic structure in two agricultural soils submitted to various types of organic management. Microb. Ecol. 2007;53:650–63.

22. Zhen Z, Liu H, Wang N, Guo L, Meng J, Ding N, et al. Effects of manure compost application on soil microbial community diversity and soil microenvironments in a temperate cropland in China. PLoS One. 2014;9:e108555.

23. Lauber CL, Ramirez KS, Aanderud Z, Lennon J, Fierer N. Temporal variability in soil microbial communities across land-use types. ISME J. 2013;7:1641–50.

24. Jansa J, Mozafar A, Anken T, Ruh R, Sanders IR, Frossard E. Diversity and structure of AMF communities as affected by tillage in a temperate soil. Mycorrhiza. 2002;12:225–34.

25. Drijber RA, Doran JW, Parkhurst AM, Lyon DJ. Changes in soil microbial community structure with tillage under long-term wheat- fallow management. Soil Biol. Biochem. 2000;32:1419–1430.

26. Alguacil M., Lumini E, Roldan A, Salinas-Garcia J, Bonafante P, Bianciotto V. The impact of tillage practices on arbuscular mycorrhizal fungal diversity in subtropical crops. Ecol. Appl. 2008;18:527–36.

27. Verbruggen E, Kiers TE. Evolutionary ecology of mycorrhizal functional diversity in agricultural systems. Evol. Appl. 2010;3:547–60.

28. Helgason BL, Walley FL, Germida JJ. Fungal and bacterial abundance in long-term no-till and intensive-till soils of the Northern Great Plains. Soil Sci. Soc. Am. J. 2009;73:120–7.

29. Wang Z, Chen Q, Liu L, Wen X, Liao Y. Responses of soil fungi to 5-year conservation tillage treatments in the drylands of northern China. Appl. Soil Ecol. 2016;101:132–40.

30. Liu J, Sui Y, Yu Z, Shi Y, Chu H, Jin J, et al. Soil carbon content drives the biogeographical distribution of fungal communities in the black soil zone of northeast China. Soil Biol. Biochem. 2015;83:29–39.

31. Lauber CL, Strickland MS, Bradford MA, Fierer N. The influence of soil properties on the structure of bacterial and fungal communities across land-use types. Soil Biol. Biochem. 2008;40:2407–15.

32. Fierer N, Jackson RB. The diversity and biogeography of soil bacterial communities. Proc. Natl. Acad. Sci. U. S. A. 2006;103:626–31.

33. Lauber CL, Hamady M, Knight R, Fierer N. Pyrosequencing-based assessment of soil pH as a predictor of soil bacterial community structure at the continental scale. Appl. Environ. Microbiol. 2009;75:5111–20.

34. Rousk J, Bååth E, Brookes PC, Lauber CL, Lozupone C, Caporaso JG, et al. Soil bacterial and fungal communities across a pH gradient in an arable soil. ISME J. 2010;4:1340–51.

35. Bulgarelli D, Schlaeppi K, Spaepen S, Ver Loren van Themaat E, Schulze-Lefert P. Structure and functions of the bacterial microbiota of plants. Annu. Rev. Plant Biol. 2013;64:807–38.

36. Jumpponen A, Johnson LC. Can rDNA analyses of diverse fungal communities in soil and roots detect effects of environmental manipulations--a case study from tallgrass prairie. Mycologia. 2005;97:1177–94.

37. Chaudhry V, Rehman A, Mishra A, Chauhan PS, Nautiyal CS. Changes in Bacterial Community Structure of Agricultural Land Due to Long-Term Organic and Chemical Amendments. Microb. Ecol. 2012;64:450–60.

38. Lupatini M, Korthals GW, de Hollander M, Janssens TKS, Kuramae EE. Soil Microbiome Is More Heterogeneous in Organic Than in Conventional Farming System. Front. Microbiol. 2017;7:10.3389/fmicb.2016.02064.

39. Verbruggen E, Röling WFM, Gamper HA, Kowalchuk GA, Verhoef HA, van der Heijden MGA. Positive effects of organic farming on below-ground mutualists : large-scale comparison of mycorrhizal fungal communities in agricultural soils. New Phytol. 2010;186:968–79.

40. Liu B, Tu C, Hu S, Gumpertz M, Ristaino JB. Effect of organic, sustainable, and conventional management strategies in grower fields on soil physical, chemical, and biological factors and the incidence of Southern blight. Appl. Soil Ecol. 2007;37:202–14.

41. Hartmann M, Widmer F. Community structure analyses are more sensitive to differences in soil bacterial communities than anonymous diversity indices. Appl. Environ. Microbiol. 2006;72:7804–12.

42. Edwards J, Johnson C, Santos-Medellín C, Lurie E, Podishetty NK, Bhatnagar S, et al. Structure, variation, and assembly of the root-associated microbiomes of rice. Proc. Natl. Acad. Sci. 2015;112:E911–20.

43. Seghers D, Wittebolle L, Top EM, Verstraete W, Siciliano S. Impact of agricultural practices on the Zea mays L. endophytic community. Appl. Environ. Microbiol. 2004;70:1475–82.

SUPPLEMENTARY FIGURES

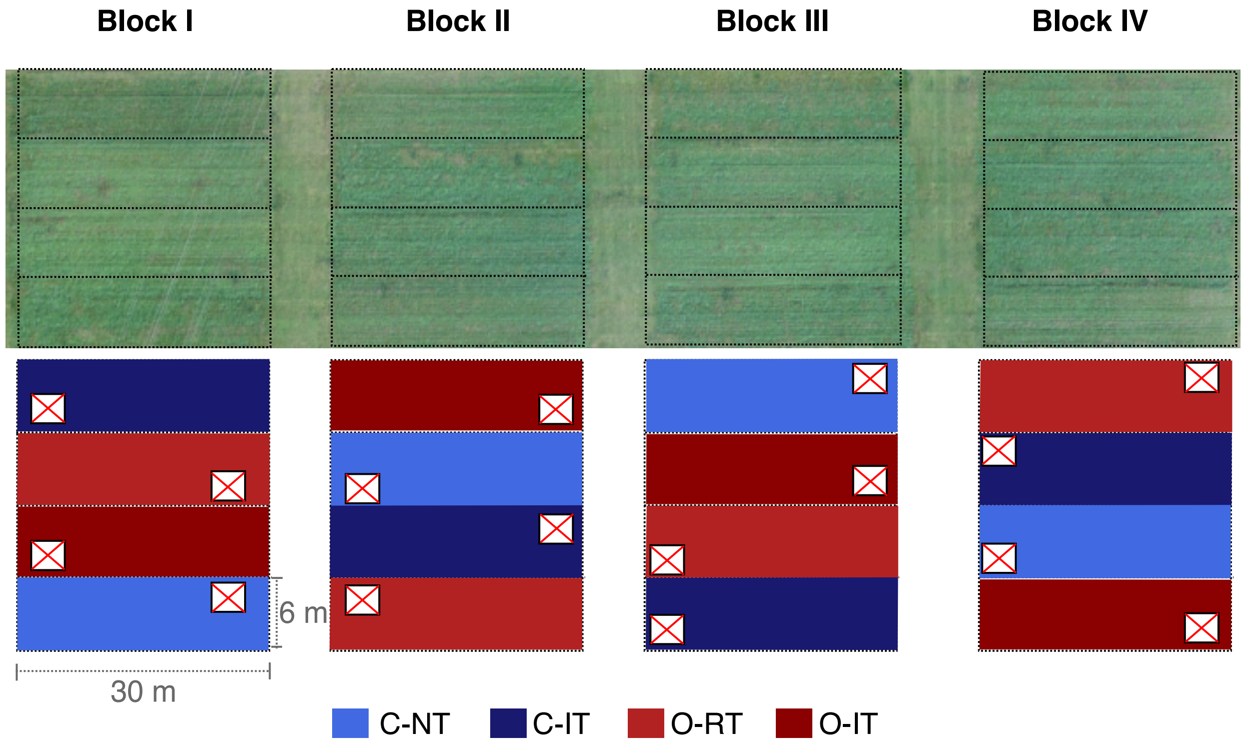

Figure S1: Experimental layout of the FAST experiment. The top panel is an aerial photograph of the four blocks at experimental site with individual plots outlined in dashed lines. The cropping system applied to each plot is indicated in the colored bottom panel. White boxes marked with X's indicate the approximate sampling location of root and soil samples within each plot.

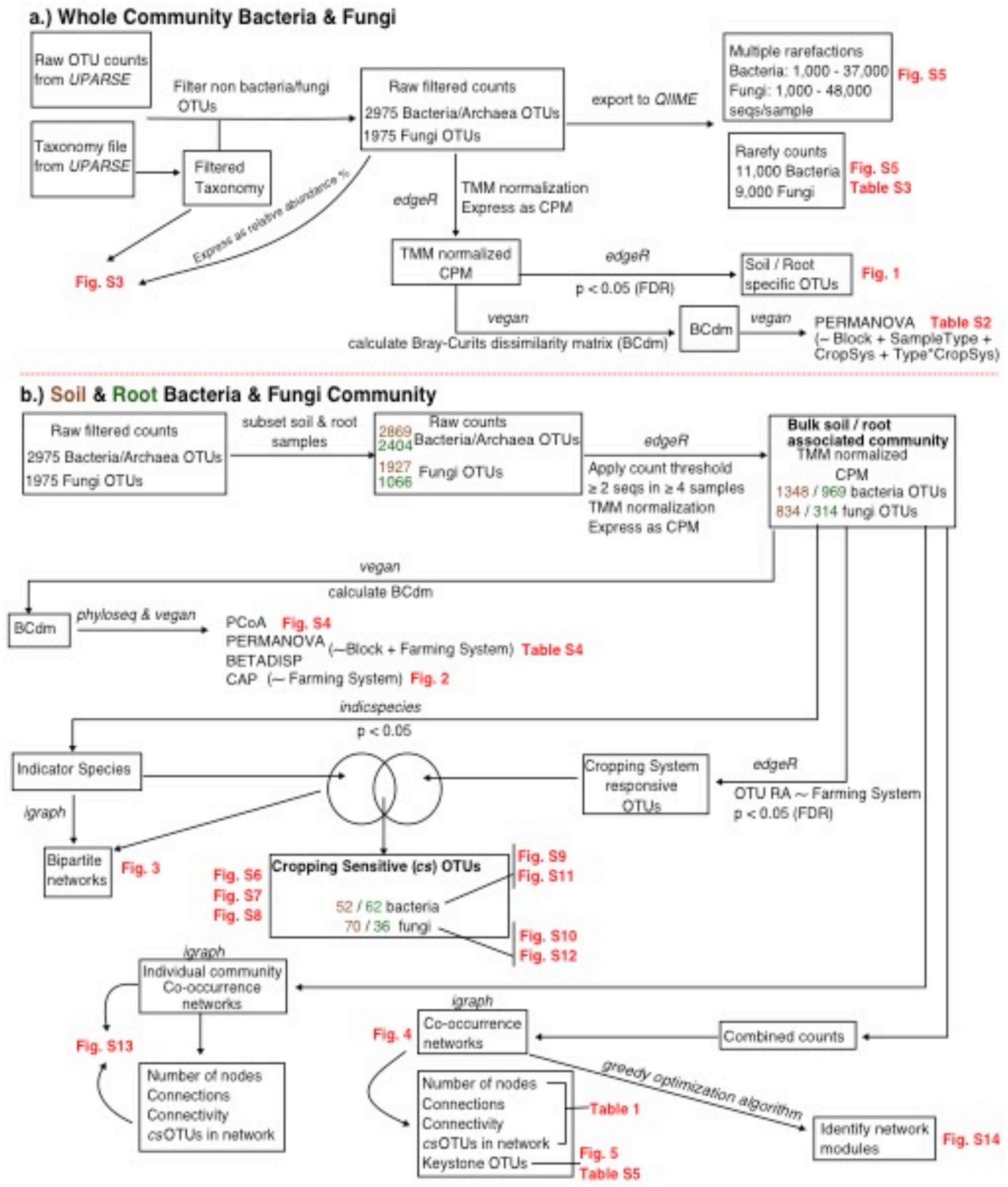

Figure S2: Schematic flow diagram of analysis steps for **(a)** whole community **(b)** soil and root bacterial and fungal communities. Numbers in brown refer to soil samples. Numbers in green refer to root samples. The figures generated as the output from each step are indicated in red.

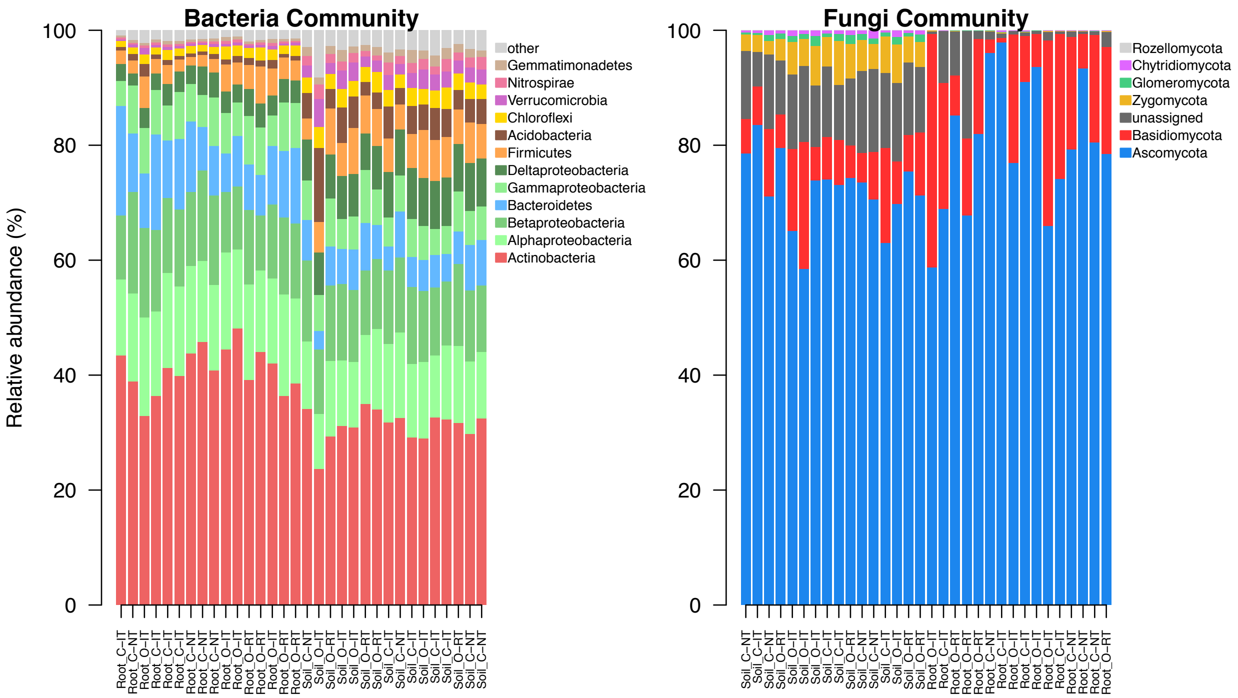

Figure S3: Taxonomic profiles of bacteria **(a)** and fungi **(b)** communities at phylum level. Bacteria phyla with relative abundances lower than 1% were summarized with 'other'. The x-axis sample order reflects a clustering by Bray-Curtis dissimilarities using the *hclust* function in R with method "average".

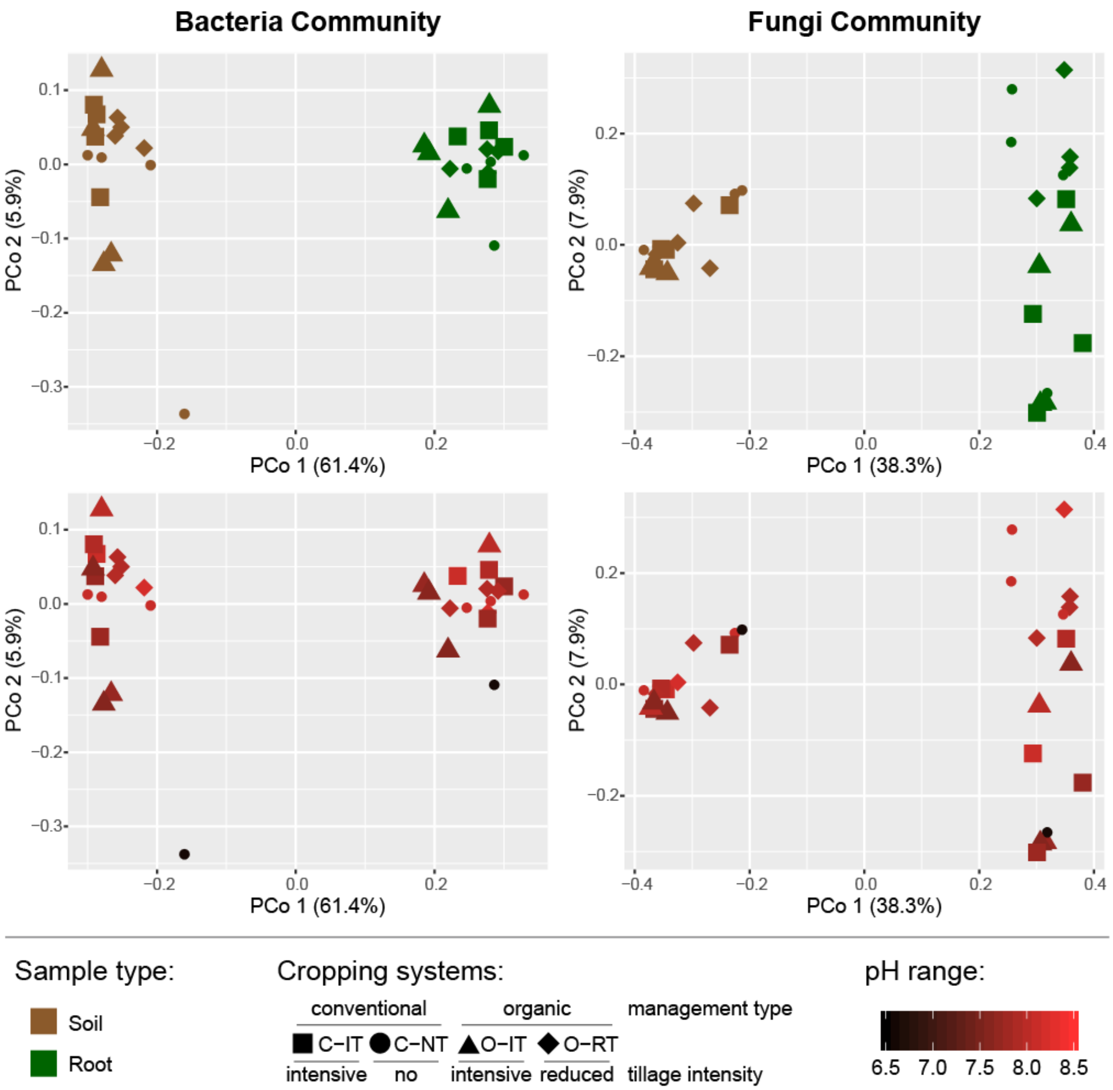

Figure S4: Unconstrained PCoA ordinations of bacteria (left) and fungi (right). Sample type presented the major driver of community variation. Percentage of variation given on each axis refers to the explained fraction of total variation in the community. Upper and lower panels are colored by sample type (root vs. soil samples) and soil pH values, respectively. Symbols refer to the different cropping systems.

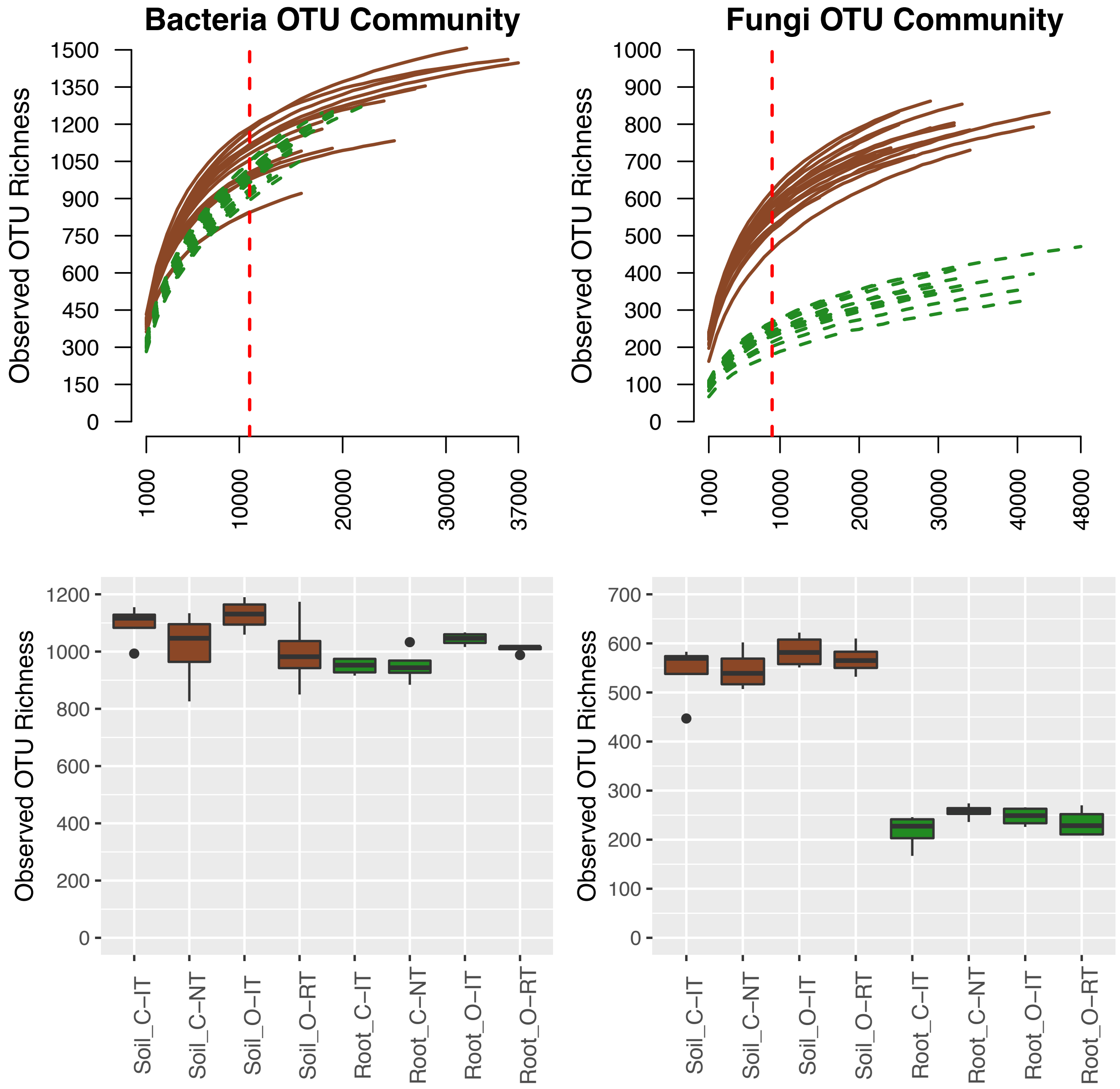

Figure S5: Rarefaction curves for bacteria and fungi observed OTU richness. Brown lines indicate soil samples, and green lines indicate root samples. The dashed red line indicates the selected rarefaction depth used to generate the box plots below each curve, 11,000 seqs/sample and 9,000 sequences per sample for bacterial and fungal communities, respectively. The boxplots show the effective OTU richness at the respective rarefaction depths for bacteria and fungi. *X* axis labels indicate the sample type and cropping system of each box, which are colored by sample type. Results of the t-tests, 2-way ANOVA and subsequent post-hoc tests, if applicable, are given in **Table S3**.

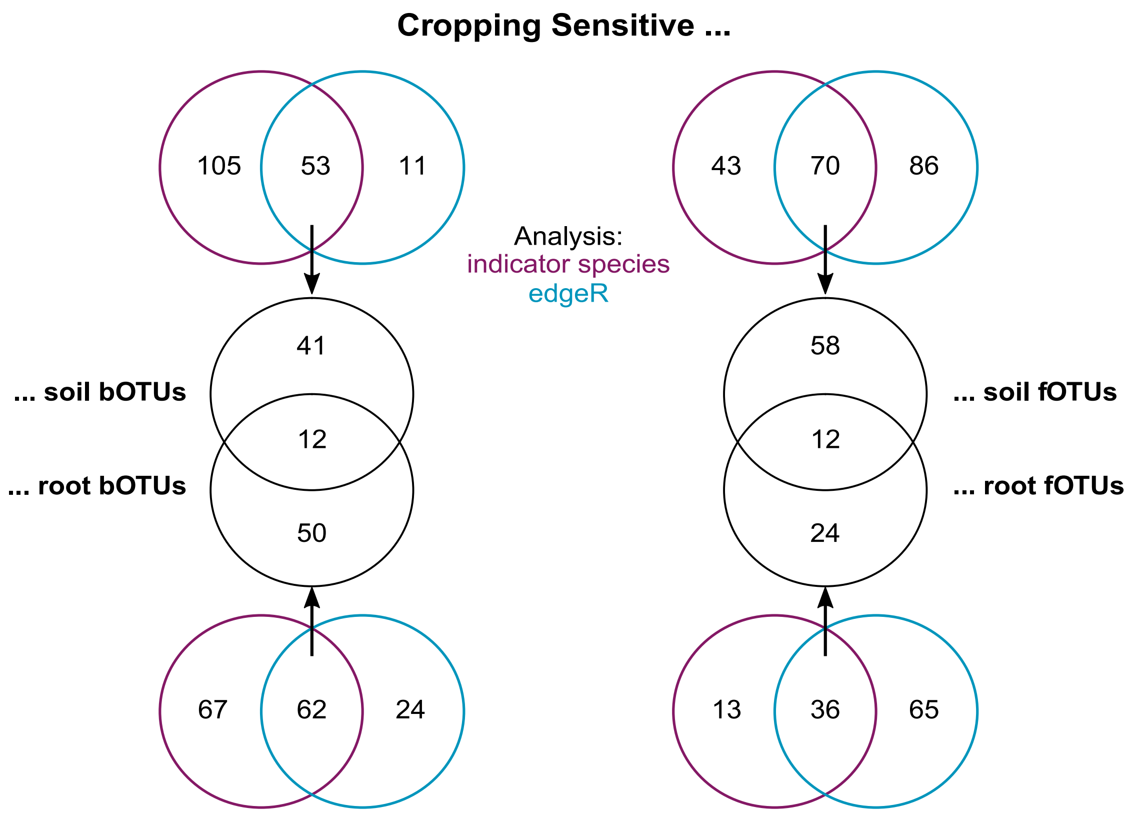

Figure S6: Defining cropping sensitive bacteria (b-) and fungi (f-) OTUs in soil and root samples. Venn diagrams show the number of OTUs responding to cropping practices identified with indicator species analysis (purple) and by edgeR (cyan). OTUs identified by both methods were defined as cropping sensitive OTUs (*cs*OTUs).

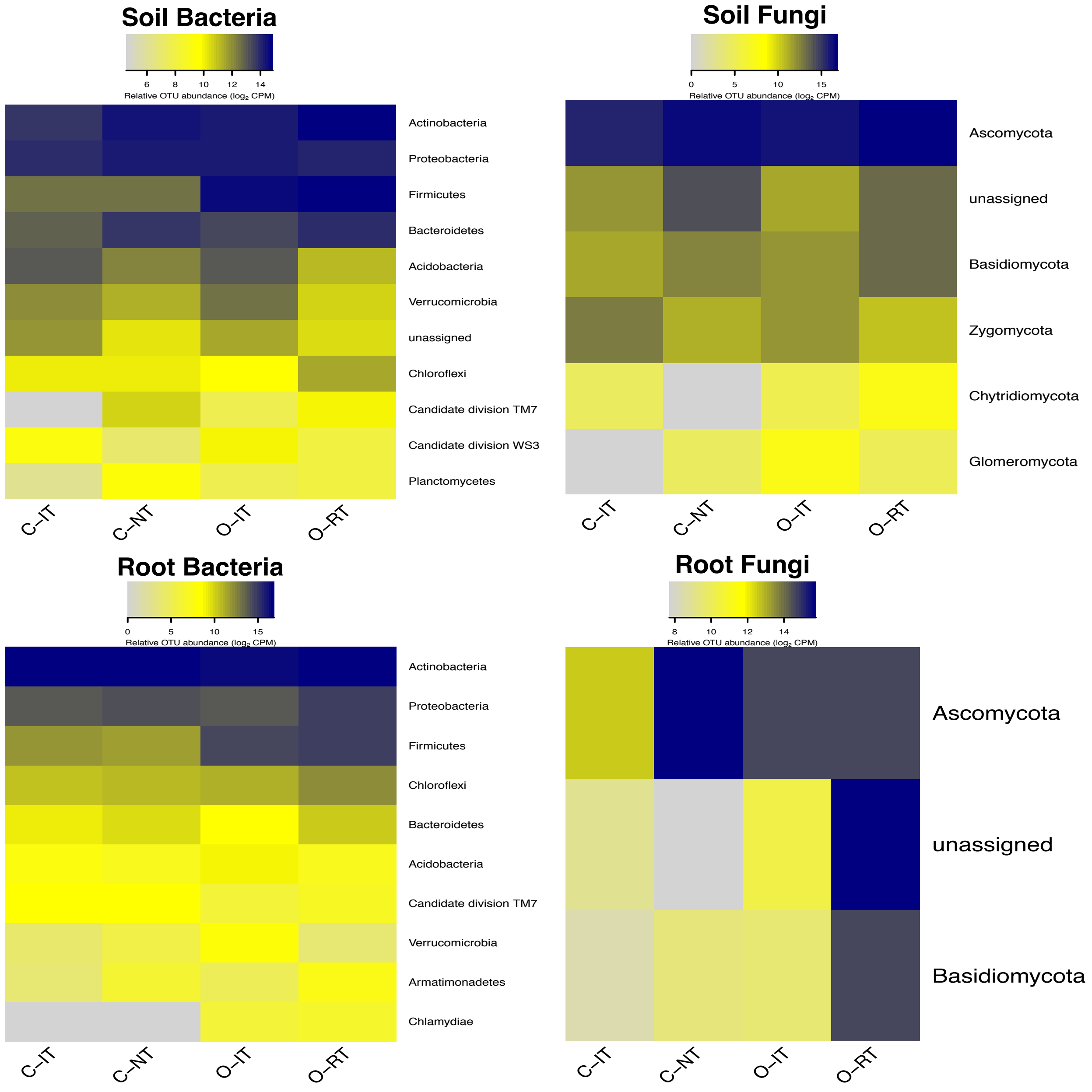

Figure S7: Mean relative abundances (counts per million, CPM; log2 scale) of cropping sensitive OTUs (as defined in **Fig. S6**, summarized at phylum level) across cropping systems for soil bacteria, soil fungi, root bacteria, and root fungi.

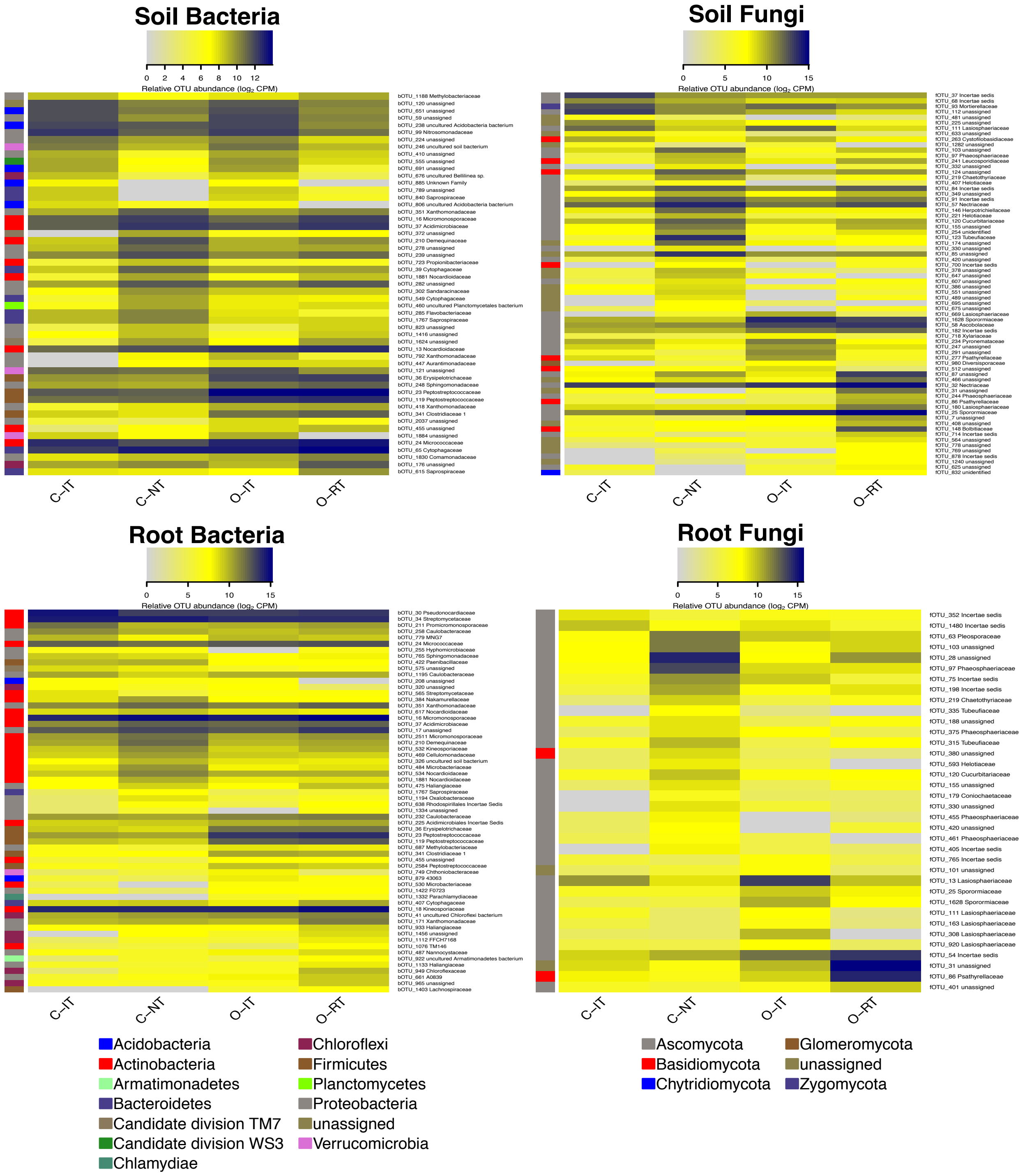

Figure S8: Mean relative abundances (counts per million, CPM; log2 scale) of cropping sensitive OTUs identified by indicator species analysis and *edgeR* (see **Fig. S6**). OTUs are labeled with their family level taxonomy assignment, with the phylum level taxonomy assignment indicated by the colored bars.

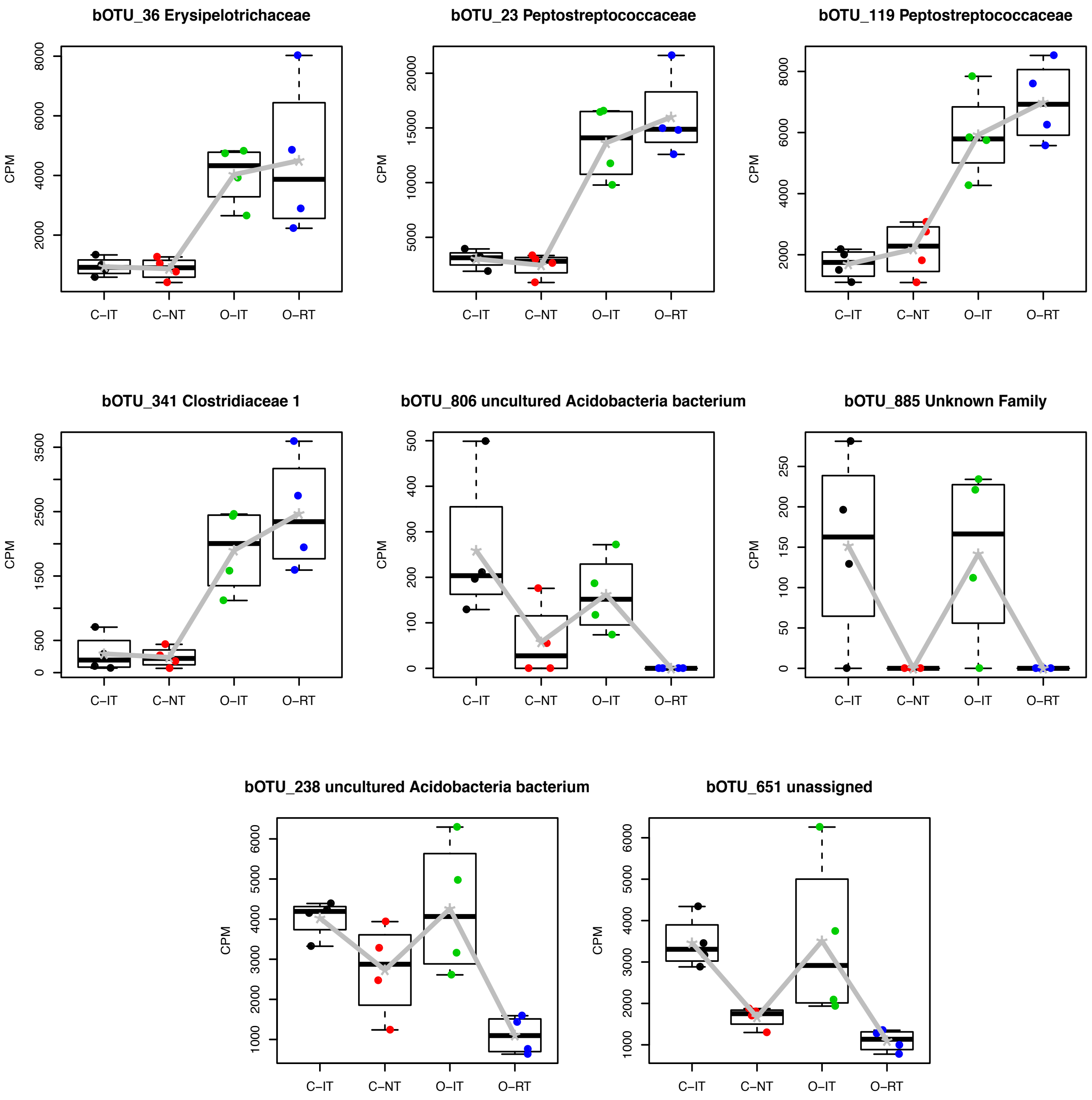

Figure S9: Relative abundances (counts per million, CPM) of abundant cropping sensitive bacteria bOTUs in soil. Means within each cropping system are indicated in gray stars.

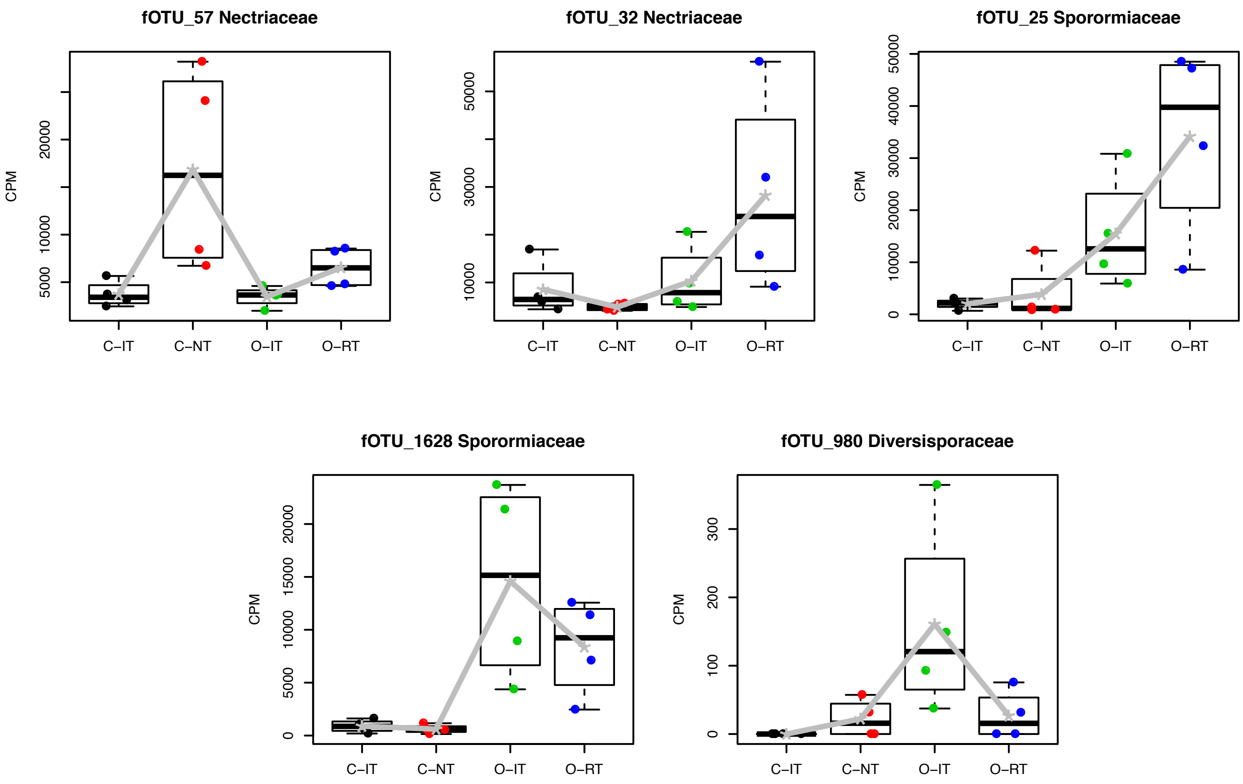

Figure S10: Relative abundances (counts per million, CPM) of abundant cropping sensitive fungi fOTUs in soil. Means within each cropping system are indicated in gray stars.

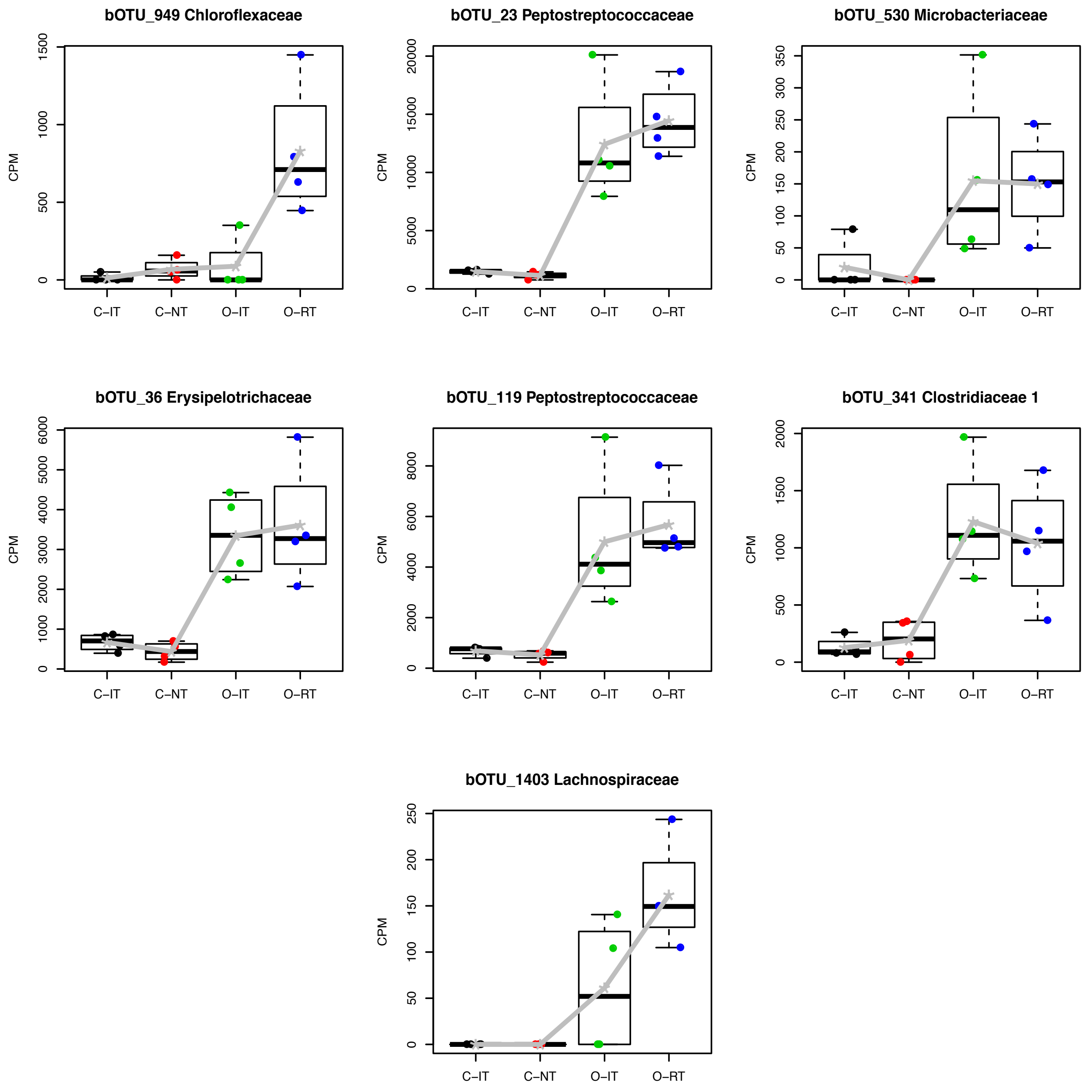

Figure S11: Relative abundances (counts per million, CPM) of abundant cropping sensitive bacteria bOTUs in roots. Means within each cropping system are indicated in gray stars.

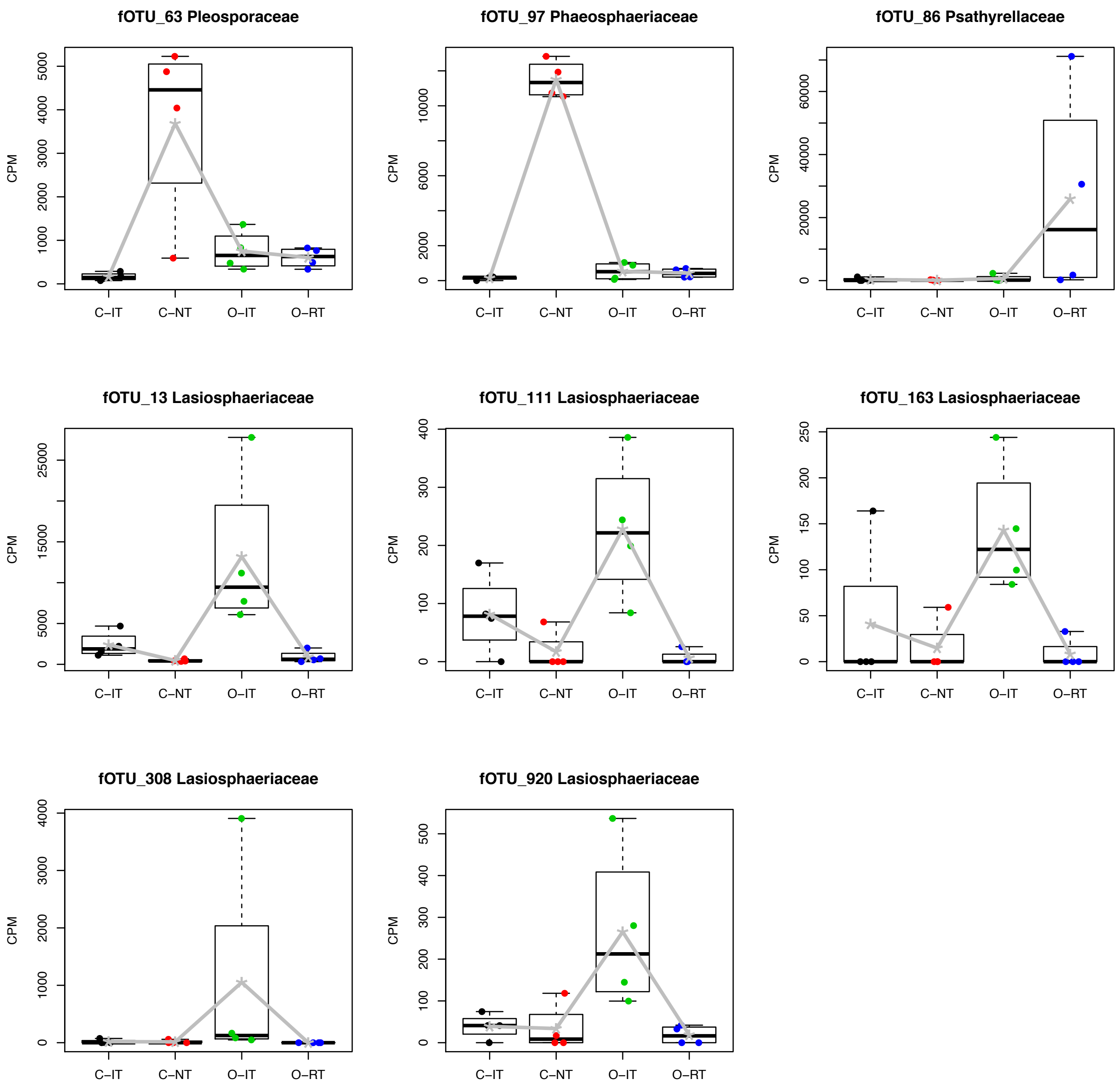

Figure S12: Relative abundances (counts per million, CPM) of abundant cropping sensitive fungi fOTUs in roots. Means within each cropping system are indicated in gray stars.

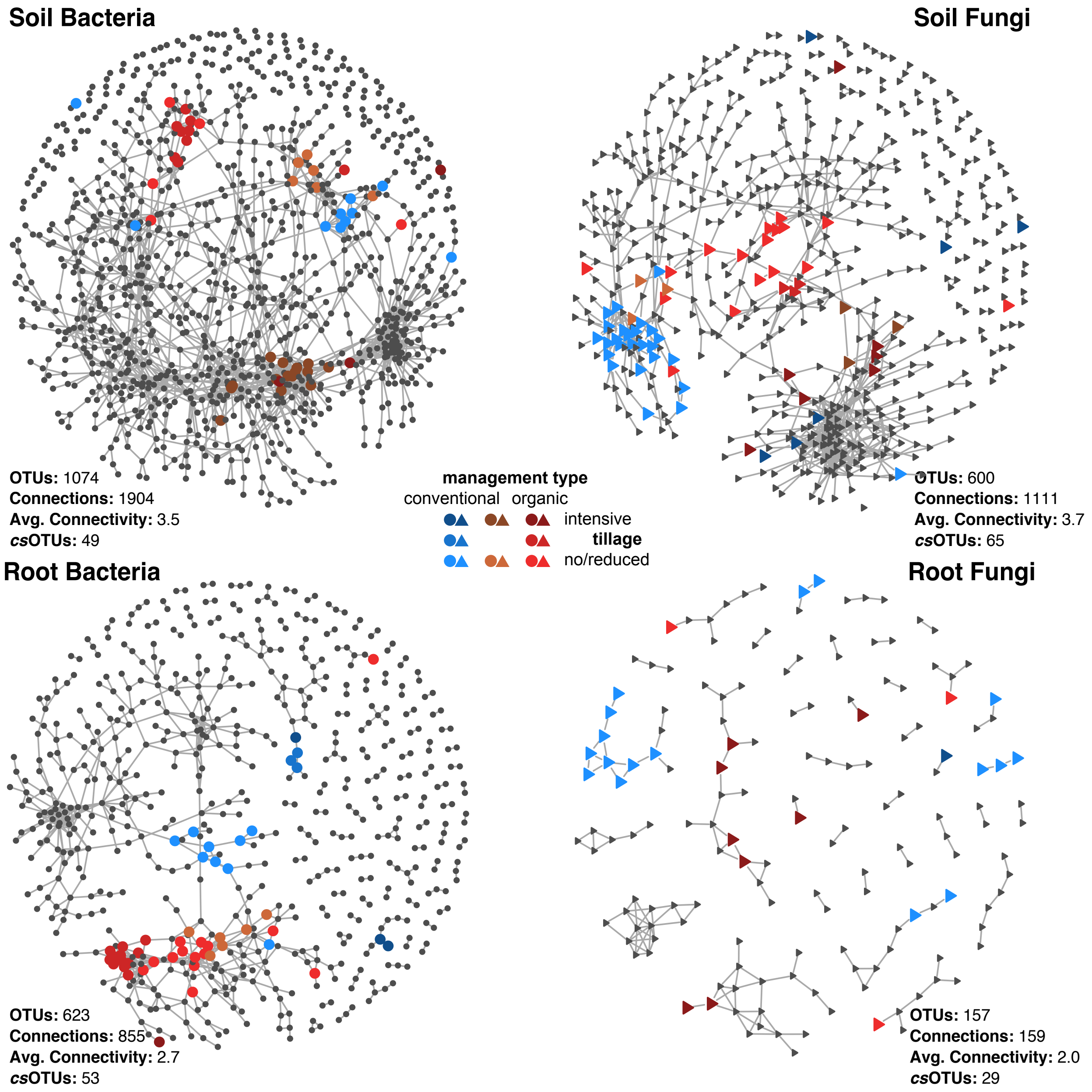

Figure S13: Co-occurrence networks visualizing significant correlations (ρ>0.7, *p<0.001*; indicated with grey lines) between OTU pairs in the soil and root bacterial and fungal communities. Circles and triangles represent bacteria and fungi OTUs, respectively. OTUs were colored by their association to the different cropping systems (as defined in **Fig. S6**, gray OTUs are insensitive to cropping practices). General network properties are indicated under each network and include: number of OTUs, number of connections, average number of connections between OTUs (avg. connectivity) and the number of *c*ropping *s*ensitive OTUs (*cs*OTUs) in the network.

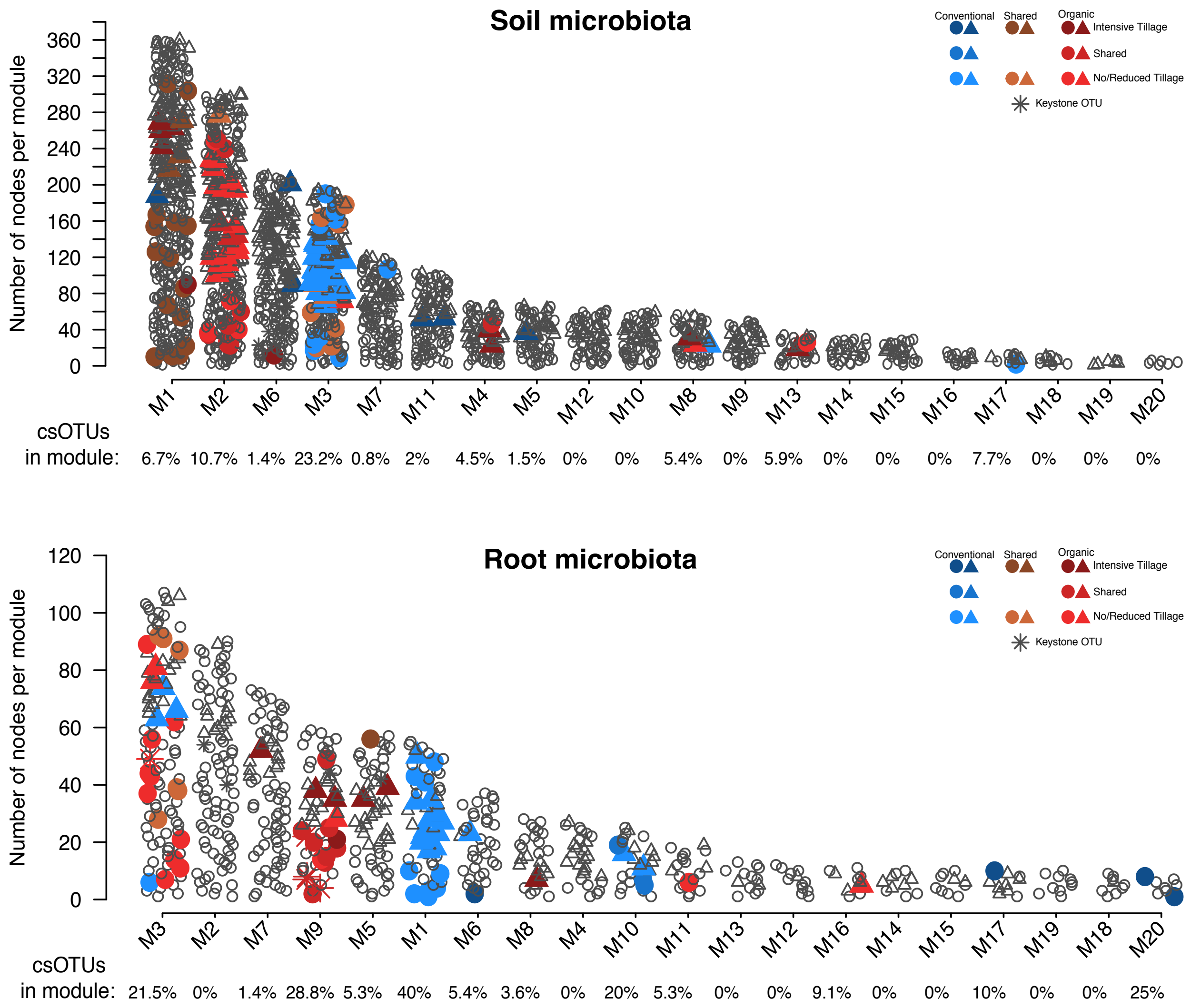

Figure S14: Defining network modules. Plots showing the number of OTUs in the top 20 most populated modules for the soil and root meta co-occurrence networks. Circles and triangles represent bacteria and fungi OTUs, respectively. OTUs were colored by their association to the different cropping systems (as defined in **Fig. S6**, gray OTUs (open symbols) are insensitive to cropping practices). Percentages on the x-axis indicate the proportion of *cs*OTUs present in each module.

SUPPLEMENTARY TABLES

Table S1: PCR cycling conditions used to generate the 16S and ITS amplicons for high-throughput sequencing.

**16S**				**ITS**			
Step	Temperature	Time	Cycles	Step	Temperature	Time	Cycles
1	94°C	2min	1x	1	94°C	5min	1x
2	94°C	30sec		2	94°C	30sec	
3	55°C	30sec	30x	3	57°C	30sec	30x
4	65°C	30sec		4	72°C	30sec	
5	65°C	10min	1x	5	72°C	7min	1x
6	15°C	hold		6	15°C	hold	

Table S2: Results of PERMANOVA testing the effects of *Block*, *Sample type* and *Cropping System* on bacterial and fungal communities. Significant effects are indicated in bold (**p<0.05*, ***p<0.01,* ****p<0.001*).

	**Bacteria**		**Fungi**	
	pseudo-F	R^2	pseudo-F	R^2
**Block** $_{(3,21)}$	1.307	0.043	0.922	0.052
**Sample type** $_{(1,21)}$	**54.665*** **	**0.602**	**19.886*** **	**0.376**
**Crop. System** $_{(3,21)}$	**2.604***	**0.086**	**1.791***	**0.102**
**Type*CropSys** $_{(3,21)}$	1.132	0.037	1.285	0.073

Table S3: Statistic testing for differences in α-diversity between root and soil samples in bacterial and fungal communities. Separate t-tests were conducted for each kingdom using a model testing for differences between sample types. Similarly, for each sample type we conducted separate ANOVAs testing the effects of *Block* and *Cropping System*. Significant effects are indicated in bold (*p<0.05, ***p<0.001). Different letters in the Tukey pairwise comparisons indicate significant differences at $p<0.05$.

	**Bacteria**		**Fungi**	
	**Soil**	**Root**	**Soil**	**Root**
**Mean ± SEM**	1058.31 ± 27.44	988.31 ± 13.24	560.25 ± 10.98	239 ± 7.06
**T-test**				
**Sample type $_{(1,30)}$**	**t=2.3***		**t=24.61*****	
**ANOVA**				
**Block $_{(3,9)}$**	F=2.22	F=1.91	F=1.25	F=0.59
**Crop. system $_{(3,9)}$**	F=1.88	**F=7.77****	F=1.25	F=2.39
				**Pairwise Comparisons**
**Cropping System**	**Mean ± SEM**	**Mean ± SEM**	**Mean ± SEM**	**Mean ± SEM**
C-IT	1095.25 ± 35.3 **a**	949.25 ± 15.18 **a**	541.25 ± 34.79 **a**	212.25 ± 16.78 **a**
C-NT	1013.25 ± 67.4 **a**	951 ± 30.75 **a**	536 ± 15.19 **a**	252 ± 8.75 **a**
O-IT	1127.75 ± 28.68 **a**	1044 ± 11.51 **b**	583.75 ± 16.91 **a**	256.25 ± 6.84 **a**
O-RT	997 ± 66.8 **a**	1009 ± 7.01 **ab**	577 ± 11.62 **a**	235.5 ± 13.91 **a**

Table S4: Results of PERMANOVA testing the effects of *Block* and *Cropping System* on bacterial and fungal communities in soil and root samples. Significant effects are indicated in bold (*$p<0.05$, **$p<0.01$, ***$p<0.001$). Different letters in the pairwise comparisons indicate significant differences at $p<0.05$ (FDR corrected). Results of BETADISP testing for differences in multivariate dispersion between cropping systems in root and soil samples in bacterial and fungal communities.

	**Soil**				**Root**			
	**Bacteria**		**Fungi**		**Bacteria**		**Fungi**	
	pseudo-F	R^2	pseudo-F	R^2	pseudo-F	R^2	pseudo-F	R^2
**Block** $_{(3,9)}$	1.09	0.18	0.73	0.14	1.02	0.17	0.95	0.17
**Crop. system** $_{(3,9)}$	**_1.85***_**	**_0.31_**	**_1.59*_**	**_0.30_**	**_2.04***_**	**_0.34_**	**_1.54**_**	**_0.28_**
**Pairwise Cropping System Comparisons**								
	C-IT (a)		*C-IT (ac)*		*C-IT (a)*		*C-IT (a)*	
	C-NT (b)		*C-NT (a)*		*C-NT (b)*		*C-NT (a)*	
	O-IT (ab)		*O-IT (b)*		*O-IT (c)*		*O-IT (a)*	
	O-RT (c)		*O-RT (c)*		*O-RT (ac)*		*O-RT (a)*	
**Multivariate homogeneity of groups dispersions**								
**Crop. system** $_{(3,12)}$	1.25		1.20		0.61		0.01	

Table S5: Keystone OTUs identified in soil and root microbial communities documented with taxonomy assignments, OTU IDs, degree of co-occurrence values, and sensitivity to cropping practices.

	Phylum	Class	Order	Family	Genus	Node	Degree	csOTU
**Soil microbial community**								
Bacteria	Actinobacteria	Thermoleophilia	Gaiellales	unassigned	unassigned	bOTU_537	44	No
	Chloroflexi	Chloroflexia	Chloroflexales	Roseiflexaceae	Roseiflexus	bOTU_443	35	No
	Proteobacteria	Alphaproteobacteria	Rhizobiales	JG34-KF-361	unassigned	bOTU_1110	52	No
				Xanthobacteraceae	Pseudolabrys	bOTU_96	57	No
		Betaproteobacteria	SC-I-84	unassigned	unassigned	bOTU_62	55	No
						bOTU_411	35	No
			TRA3-20			bOTU_180	41	No
		Gammaproteobacteria	Xanthomonadales			bOTU_331	44	No
	Verrucomicrobia	OPB35 soil group	unassigned			bOTU_637	38	No
						bOTU_897	38	No
Fungi	Ascomycota	Sordariomycetes	Chaetosphaeriales	Chaetosphaeriaceae	Chaetosphaeria	fOTU_278	42	No
		Sordariomycetes	Chaetosphaeriales	Chaetosphaeriaceae	unassigned	fOTU_71	42	No
		Sordariomycetes	Sordariales	unassigned		fOTU_641	43	No
	Basidiomycota	Tremellomycetes	unassigned			fOTU_831	57	No
	unassigned	unassigned				fOTU_494	70	No
						fOTU_208	46	No
						fOTU_450	46	No
						fOTU_201	38	No
	Zygomycota	Incertae sedis	Mortierellales	Mortierellaceae	Mortierella	fOTU_337	43	No
**Root microbial community**								
Bacteria	Acidobacteria	Acidobacteria	Subgroup 2	unassigned	unassigned	bOTU_1141	18	No
	Actinobacteria	Actinobacteria	Micrococcales	Microbacteriaceae	Leucobacter	bOTU_530	18	**Yes**
		Thermoleophilia	Gaiellales	unassigned	unassigned	bOTU_1091	17	No
	Chloroflexi	Chloroflexia	Chloroflexales	Chloroflexaceae	Chloronema	bOTU_949	17	**Yes**
	Firmicutes	Bacilli	Bacillales	unassigned	unassigned	bOTU_267	21	No
		Clostridia	Clostridiales	Peptostreptococcaceae	Incertae Sedis	bOTU_23	17	**Yes**
						bOTU_119	16	**Yes**
		Erysipelotrichia	Erysipelotrichales	Erysipelotrichaceae	Turicibacter	bOTU_36	16	**Yes**
	Proteobacteria	Alphaproteobacteria	Rhizobiales	unassigned	unassigned	bOTU_54	16	No
